# A stable covalent organic framework for photocatalytic carbon dioxide reduction†
†Electronic supplementary information (ESI) available. See DOI: 10.1039/c9sc03800k


**DOI:** 10.1039/c9sc03800k

**Published:** 2019-11-21

**Authors:** Zhiwei Fu, Xiaoyan Wang, Adrian M. Gardner, Xue Wang, Samantha Y. Chong, Gaia Neri, Alexander J. Cowan, Lunjie Liu, Xiaobo Li, Anastasia Vogel, Rob Clowes, Matthew Bilton, Linjiang Chen, Reiner Sebastian Sprick, Andrew I. Cooper

**Affiliations:** a Department of Chemistry and Materials Innovation Factory , University of Liverpool , 51 Oxford Street , Liverpool L7 3NY , UK . Email: ssprick@liverpool.ac.uk ; Email: aicooper@liverpool.ac.uk ; Email: lchen@liverpool.ac.uk; b Stephenson Institute for Renewable Energy , University of Liverpool , Chadwick Building, Peach Street , Liverpool L69 7ZF , UK; c Leverhulme Research Centre for Functional Materials Design , Materials Innovation Factory and Department of Chemistry , University of Liverpool , Oxford Street , Liverpool L7 3NY , UK; d Imaging Centre at Liverpool , University of Liverpool , Liverpool L69 3GL , UK

## Abstract

Photocatalytic conversion of CO_2_ into fuels is an important challenge for clean energy research and has attracted considerable interest. Here we show that tethering molecular catalysts—a rhenium complex, [Re(bpy)(CO)_3_Cl]—together in the form of a crystalline covalent organic framework (COF) affords a heterogeneous photocatalyst with a strong visible light absorption, a high CO_2_ binding affinity, and ultimately an improved catalytic performance over its homogeneous Re counterpart. The COF incorporates bipyridine sites, allowing for ligation of the Re complex, into a fully π-conjugated backbone that is chemically robust and promotes light-harvesting. A maximum rate of 1040 μmol g^–1^ h^–1^ for CO production with 81% selectivity was measured. CO production rates were further increased up to 1400 μmol g^–1^ h^–1^, with an improved selectivity of 86%, when a photosensitizer was added. Addition of platinum resulted in production of syngas, hence, the co-formation of H_2_ and CO, the chemical composition of which could be adjusted by varying the ratio of COF to platinum. An amorphous analog of the COF showed significantly lower CO production rates, suggesting that crystallinity of the COF is beneficial to its photocatalytic performance in CO_2_ reduction.

## Abstract 2

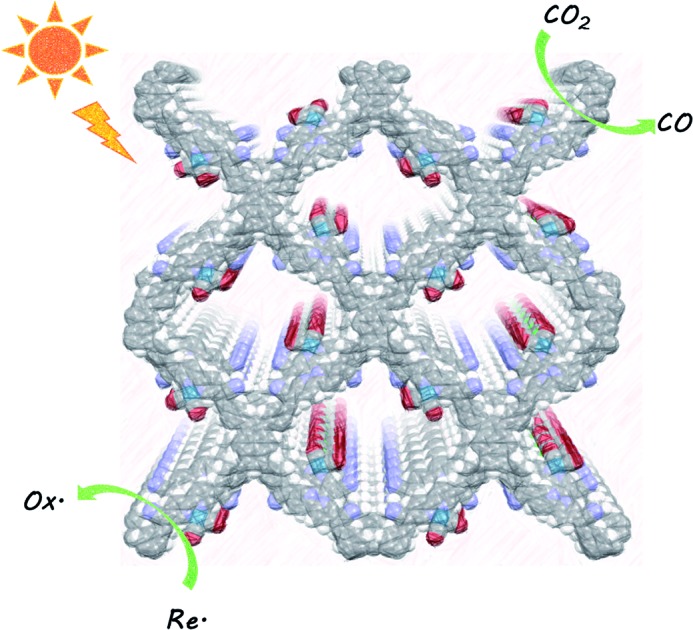
A metal-decorated alkene-linked covalent organic framework is a robust, selective photocatalyst for CO_2_ reduction.

## Introduction

There is now little doubt that the human production of CO_2_ has contributed to climate change, which will affect life on earth.^1^ One potential approach to reducing CO_2_ emissions is its conversion into value-added products using solar energy.^2^,^3^ A range of inorganic semiconductors has been investigated as photocatalysts for CO_2_ reduction, such as TiO_2_,^4^ ZrO_2_,^5^ In_2_O_3_,^6^ CdS,^7^ or ZnGa_2_O_4_.^8^ Unfortunately, many inorganic photocatalysts either have unsuitably aligned conduction/valence band positions or relatively large band gaps, hence limiting visible light absorption. By contrast, the band gap in organic semiconductors can be tuned readily through the incorporation of a diverse range of monomers.^9^–^11^ In recent years, porous organic materials such as carbon nitrides,^12^–^14^ conjugated microporous polymers (CMPs),^15^,^16^ covalent triazine-based frameworks (CTFs)^17^ and hyper-crosslinked polymers (HCPs)^18^ have been studied for photocatalytic CO_2_ reduction. Those organic materials are typically amorphous; by contrast, covalent organic frameworks (COFs) can combine porosity with crystallinity.^19^–^23^ COFs have been investigated as photocatalysts for water splitting,^24^,^25^ and for electrocatalytic CO_2_ reduction.^26^,^27^ These materials also have potential for direct photocatalytic CO_2_ reduction: for example, an azine-based COF, N_3_-COF, was shown to exhibit gas phase photocatalytic CO_2_ reduction.^28^ Likewise, a 2D imine triazine-COF loaded with rhenium^29^ and a β-ketoenamine-linked COF decorated with both nickel and a light-absorbing dye^30^ were studied for the same reaction. All of these COFs have limited effective conjugation lengths in the 2D plane of the framework because they are based on imine, azine, or β-ketoenamine-linkers. This results in blue-shifted absorption on-sets, which limit the ability of the materials to absorb visible light.^31^,^32^

**Fig. 1:**
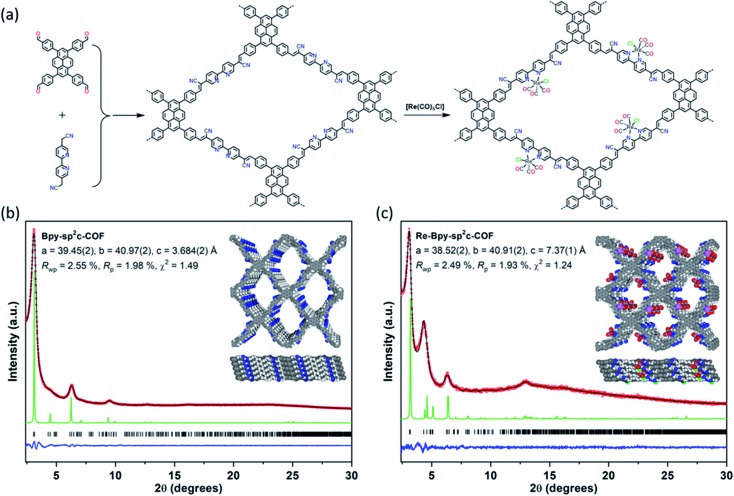
(a) Synthesis of **Bpy-sp^2^c-COF** and **Re-Bpy-sp^2^c-COF**. Conditions for **Bpy-sp^2^c-COF**: KOH (4 M) 1,2-dichlorobenzene and 1-butanol (1 : 1 mixture), 120 °C, 72 hours; (b) and (c) PXRD patterns of **Bpy-sp^2^c-COF** (b) and **Re-Bpy-sp^2^c-COF** (c) obtained experimentally (red circles), simulated from the eclipsed AA-stacking mode (green), profiles calculated from Le Bail fitting (black) and residual (blue). Reflection positions are shown by tick marks. Structural models for **Bpy-sp^2^c-COF** (b) and **Re-Bpy-sp^2^c-COF** (c) with eclipsed AA stacking patterns, shown parallel to the pore channel along the crystallographic *c* axis (top) and parallel to the layers (bottom).

Here, we used Knoevenagel condensation (Fig. 1a) such that olefins become the COF linkers.^31^,^33^,^34^ Our aim was to increase the conjugation length in the framework and hence, perhaps, to improve the performance of these materials for CO_2_ reduction. The cyanovinyl-groups as a result of the Knoevenagel condensation have been shown to be beneficial for CO_2_ uptake^35^ which might increase the efficiency of CO_2_ reduction. The COF was loaded with [Re(CO)_5_Cl] giving a heterogeneous analogue of the well-studied homogeneous catalyst [Re(bpy)(CO)_3_Cl] with enhanced stability.^36^

## Results and discussion

We synthesized a two-dimensional (2D) sp^2^c-COF *via* the Knoevenagel condensation of 1,3,6,8-tetrakis(4-formylphenyl)pyrene (TFPPy) and 5,5′-bis(cyanomethyl)-2,2′-bipyridine in 1,2-dichlorobenzene and 1-butanol at 120 °C (Fig. 1a). The powder X-ray diffraction (PXRD) pattern of **Bpy-sp^2^c-COF** (Fig. 1b and S9†) is in good agreement with the profile predicted for the eclipsed (AA) stacked structure (Fig. 1b, inside). Diffraction peaks are observed at 3.1°, 4.5°, 6.2°, and 9.5°, corresponding to the (110), (200), (220), and (330) reflections, suggesting that **Bpy-sp^2^c-COF** has uniform 1D diamond-shaped pores.

Nitrogen sorption experiments were performed at 77 K and the Brunauer–Emmett–Teller surface area (SA_BET_) for **Bpy-sp^2^c-COF** was calculated to be 432 m^2^ g^–1^. This SA_BET_ is lower than that predicted for the atomistic model of a perfectly crystalline structure (2041 m^2^ g^–1^), but this is commonly observed for sp^2^c-COFs which typically have surface areas ranging from 322 m^2^ g^–1^ for sp^2^c-COF-2 (ref. 37) up to 692 m^2^ g^–1^ for sp^2^c-COF.^31^

**Fig. 2:**
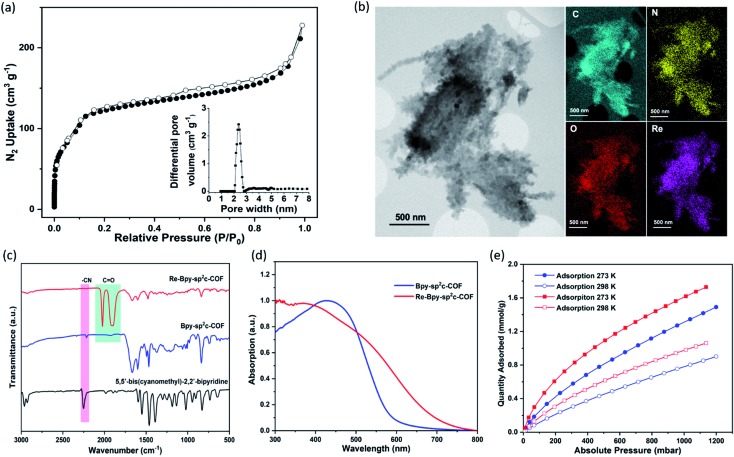
(a) N_2_ Adsorption (filled dots) and desorption (open dots) isotherm profiles of **Bpy-sp^2^c-COF** measured at 77 K. Inset: profile of the calculated pore size distribution of **Bpy-sp^2^c-COF**; (b) STEM images and EDX mapping of **Re-Bpy-sp^2^c-COF**; (c) FT-IR Spectra of **Bpy-sp^2^c-COF**, **Re-Bpy-sp^2^c-COF** and 5,5′-bis(cyanomethyl)-2,2′-bipyridine; (d) Solid-state reflectance UV-vis spectra; (e) CO_2_ adsorption isotherms of **Bpy-sp^2^c-COF** (blue) and **Re-Bpy-sp^2^c-COF** (red).

The pore size distribution profile shows a narrow pore size distribution with a pore width of 2.4 nm (Fig. 2a, inset curve), further supporting an AA stacking sequence that is predicted to have a pore size of 2.4 nm. Fourier-transform infrared (FT-IR) spectroscopy shows a distinct peak at 2217 cm^–1^ relating to a –C≡N vibration band, indicating the formation of **Bpy-sp^2^c-COF** (Fig. 2c).

Re-complexes for photocatalytic CO_2_ reduction were first reported in 1983,^38^ and they are well studied owing to their high efficiency and selectivity for CO formation.^39^,^40^ Here, we used the bipyridine sites in **Bpy-sp^2^c-COF** to ligate [Re(CO)_5_Cl] to give **Re-Bpy-sp^2^c-COF**. The PXRD pattern of the Re-incorporated COF (Fig. 1c and S10†) shows peaks at 3.1°, 4.5°, 6.2°, 12.9°, corresponding to (110), (200), (220), (–221) reflections predicted for the Re-loaded, AA-stacked model (Fig. 1c).

The BET surface area (SA_BET_) for **Re-Bpy-sp^2^c-COF** was calculated to be 323 m^2^ g^–1^ (Fig. S38†). Scanning transmission electron microscopy (STEM) and energy-dispersive X-ray spectroscopy (EDX) mapping images (Fig. 2b) show a uniform distribution of C, N, O, and Re in **Re-Bpy-sp^2^c-COF**, further suggesting that the Re moiety has been incorporated throughout the structure of COFs. Inductively coupled plasma-optical emission spectrometry (ICP-OES) measurements show that 18 wt% of Re has been incorporated into the material. This ratio corresponds to ligation of half of the bipyridine sites by the Re complex, which was also the ratio in the atomistic model built to represent **Re-Bpy-sp^2^c-COF** (Fig. 1c). The FT-IR spectrum for the Re-modified COF (Fig. 2c) shows new peaks at 1900 cm^–1^, 1917 cm^–1^, 2024 cm^–1^, corresponding to the CO-stretching bands of the incorporated [Re(CO)_3_Cl] complex.^29^,^36^ UV-visible diffuse reflectance spectra (Fig. 2d) show a red-shift of the absorption edge from 589 nm to 694 nm for **Re-Bpy-sp^2^c-COF** compared to **Bpy-sp^2^c-COF**. Finally, X-ray photoelectron spectra for Re 4f, Cl 2p and N 2s regions look very similar for **Re-Bpy-sp^2^c-COF** compared to the molecular catalyst [Re(bpy)(CO)_3_Cl] indicating that complexation of Re is similar in both cases (Fig. S40†).

We next studied the CO_2_ uptake for this material up to 1200 mbar at both 273 and 298 K. **Re-Bpy-sp^2^c-COF** adsorbs 1.7 mmol g^–1^ CO_2_ at 273 K and 1.1 mmol g^–1^ at 298 K (Fig. 2e). **Re-Bpy-sp^2^c-COF** has a high isosteric heat of adsorption (31 kJ mol^–1^), showing that the COF has good affinity toward CO_2_ (Fig. S19†).

Photocatalytic CO_2_ reduction experiments were conducted in a quartz flask under 1 atmosphere CO_2_ in a mixture of acetonitrile (MeCN) and triethanolamine (TEOA) in 30 : 1 ratio and under visible light illumination (*λ* > 420 nm, 300 W Xe light source). TEOA acts as the sacrificial electron donor and proton source, while MeCN is used to disperse the catalyst. Over a total of 17.5 hours irradiation under visible light (Fig. S16†), **Re-Bpy-sp^2^c-COF** produced CO with a rate of 1040 μmol g^–1^ h^–1^ and 81% selectivity over H_2_, which equals to a TON of 18.7 for CO, outperforming its homogeneous counterpart under the same conditions, which is deactivated after 3 hours with a TON of 10.3 (Fig. S16†).

An apparent quantum yield (AQY) of 0.5% was measured at 420 nm for CO production. The small amount of H_2_ possibly originates from competing proton reduction of water traces in the TEOA or oxidative dehydrogenation of TEOA.^13^ In the absence of the Re-complex, **Bpy-sp^2^c-COF** only generated trace amounts of CO while H_2_ was not detected. No other liquid phase products, *i.e.* HCOOH and methanol, were observed.

The combination of CO_2_ conversion rate and CO/H_2_ selectivity of **Re-Bpy-sp^2^c-COF** compares favorably with other reported COFs (see Table S3†). For example, a rhenium modified 2D imine triazine-COF produced around 750 μmol g^–1^ h^–1^ CO with 98% selectivity,^29^ and a β-ketoenamine-linked COF modified with nickel, plus the use of an additional dye, gave a CO production rate of 811 μmol g^–1^ h^–1^ CO with 96% selectivity.^30^ These comparisons should be made with caution, though, since photocatalytic rates also depend strongly on the precise experimental set-up that is used.^41^

**Fig. 3:**
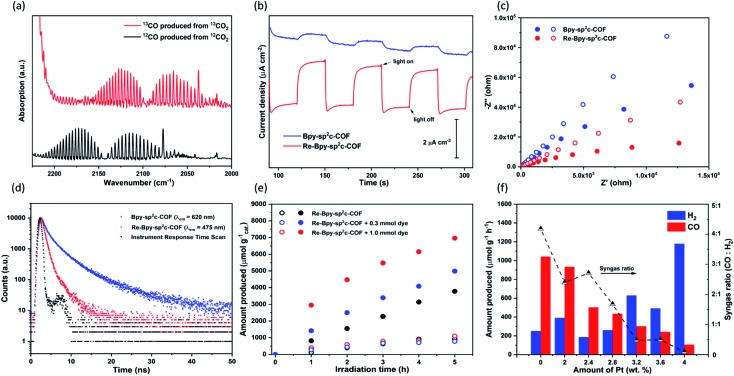
(a) FT-IR Spectra of ^13^CO produced in the photoreduction of ^13^CO_2_ (b) transient photocurrent response at 0.5 V *vs.* RHE under intermittent light irradiation for **Bpy-sp^2^c-COF** and **Re-Bpy-sp^2^c-COF**; (c) Nyquist plots of **Bpy-sp^2^c-COF** (blue) and **Re-Bpy-sp^2^c-COF** (red) at a voltage of 0.5 V *vs.* RHE under dark (open dots) and light irradiation (closed dots); (d) time-correlated single-photon counting experiments for **Bpy-sp^2^c-COF** and **Re-Bpy-sp^2^c-COF** in MeCN/TEOA (30/1) solution purged with CO_2_ (*λ*_exc_ = 405 nm); (e) CO (closed dots) and H_2_ (open dots) production using visible light (*λ* > 420 nm, 300 W Xe light source) for **Re-Bpy-sp^2^c-COF** and **Re-Bpy-sp^2^c-COF** with dye (1 mg catalyst or 1 mg catalyst with 0.3 mmol or 1.0 mmol dye in 5 mL solvent with ratio of MeCN/TEOA = 30/1); (f) photocatalytic syngas generation of Pt modified **Re-Bpy-sp^2^c-COF** under visible light irradiation (*λ* > 420 nm, 300 W Xe light source).

Control experiments were carried out to confirm that the source of the CO generated is indeed a photocatalytic process (Table S2†). Under an argon atmosphere in absence of CO_2_, **Re-Bpy-sp^2^c-COF** generated 14.9 μmol g^–1^ h^–1^ CO and 285.3 μmol g^–1^ h^–1^ H_2_. The small amount of CO produced is possibly a result of decomposition of organic residues during photocatalysis^1^ or decomposition of TEOA as an ineffective side-reaction.^13^ No gas production was observed in the dark or in the absence of hole scavenger. Experiments with isotopically labelled ^13^CO_2_ resulted in the formation of ^13^CO, strongly suggesting that CO_2_ was the source of the produced CO (Fig. 3a).

**Re-Bpy-sp^2^c-COF** appears to be stable under the photocatalysis conditions, evident from the post-illumination FT-IR spectra (Fig. S7†) of the sample after 17.5 hours of continuous visible light illumination (*λ* > 420 nm, 300 W Xe light source). The material also retains most of its crystallinity and PXRD patterns show that some order is retained after photolysis for 17.5 hours (Fig. S11†). This shows that the material has very good stability compared to other previous reports.^29^,^30^ When the run was extended to a total of 50 hours a further loss of crystallinity is observed (Fig. S43 and S44†) along a loss of activity, highlighting that stability is still one of the important challenges in the field. Nevertheless, it seems that in making a heterogeneous analogue of [Re(bpy)(CO)_3_Cl] an increase in stability is observed (Fig. S43†), possibly by preventing the formation of the dimer of the Re-complex^36^ which can occur with Re(bpy)CO_3_Cl in solution, or by the shielding of the Re-centre within the COF structure from photodecomposition side reactions.^42^

Photoelectrochemical experiments were conducted using FTO glass as a photocathode in 0.1 M Na_2_SO_4_ solution (Fig. 3b). All samples were tested at a constant voltage of 0.5 V *vs.* reversible hydrogen electrode (RHE). The photocurrent of **Re-Bpy-sp^2^c-COF** photocathode was about 2 μA cm^–1^, which was more than four times higher than a Re-free **Bpy-sp^2^c-COF** photoanode. Additionally, Nyquist plots (Fig. 3c) showed the arc radii for **Bpy-sp^2^c-COF** and **Re-Bpy-sp^2^c-COF** under irradiation were smaller than those in dark, verifying that charge carriers were generated in **Bpy-sp^2^c-COF** and **Re-Bpy-sp^2^c-COF** under irradiation. The Nyquist plots of **Re-Bpy-sp^2^c-COF** under irradiation have smaller semicircles than those of **Bpy-sp^2^c-COF**. Both measurements taken together show that the Re bearing material acts as a better photo-electro catalyst indicating that the material is better at separating and transferring charges, which is also in line with computational predictions (*vide infra*).

We then went on to use emission spectroscopy to study the mechanism of the photocatalysis for the Re loaded **Bpy-sp^2^c-COF**. The photocatalyst **Bpy-sp^2^c-COF** in acetonitrile suspension shows the presence of two emissive states, with *λ*_max_ at 475 and 640 nm. The excitation spectrum shows the 640 nm emission arises from a broad range of absorption bands in the UV/vis spectrum (from 300 to 500 nm), in contrast the sharp emission band centred at 475 nm is a result of excitation into a single band at 390 nm (Fig. S20†). Time-correlated single-photon counting (TCSPC) measurements show the lifetime of the 640 nm emissive state of **Bpy-sp^2^c-COF** is insensitive to the TEOA scavenger (Table S1 and Fig. S21†) and the emission yield is also unchanged (Fig. S20 and S21†). In contrast the 475 nm emission lifetime (2.48 ns to 0.72 ns) and yield is very sensitive to the presence of the TEOA electron donor (Fig. S20 and S21†), indicating that reductive quenching of this excited state can occur. Previous studies on closely related sp^2^c pyrene COFs have also reported the presence of two emissive states for COF samples.^37^ Therein, emission at 640 nm was attributed to the presence of a delocalised excited state across both pyrene and the sp^2^-carbon backbone on the basis of the significantly red-shifting of the emission when compared to that typically measured for excimer state of pyrene systems alone (*ca.* 480 nm). Interestingly following exfoliation of the COF, a second emission at *ca.* 468 nm was observed, proposed to be due to exfoliated COF where the removal of the π–π stacking force allows twisting of the structure and a loss of conjugation across the backbone. Here, the samples are sonicated prior to use and a similar assignment is also proposed.

Addition of the Re catalytic site to the COF framework leads to a marked change in the measured photophysical behaviour. With **Re-Bpy-sp^2^c-COF** a single emissive state (*λ*_max_ = 475 nm), proposed to be due exfoliated COF material remains. Such an assignment is in-line with the noted insensitivity of emission at this wavelength to the presence of the Re centre as the loss of conjugation of the exfoliated structure may be expected to prevent efficient electron or energy transfer from the COF framework to the Re centre. Significantly the delocalised COF excited 640 nm emissive state of **Bpy-sp^2^c-COF** is completely absent in the **Re-Bpy-sp^2^c-COF** (Fig. S22†). The quenching of the emission by the Re centre indicates possible electron transfer from the COF backbone to the catalytically active Re complex. The assignment of the sensitisation of the catalytic centre following photon absorption by the COF framework is supported by the DFT calculations below and the good agreement between the wavelength dependent CO measurement (Fig. S35†) and the excitation spectrum of the 640 nm emission of the **Bpy-sp^2^c-COF** sample (Fig. S20†).

**Fig. 4:**
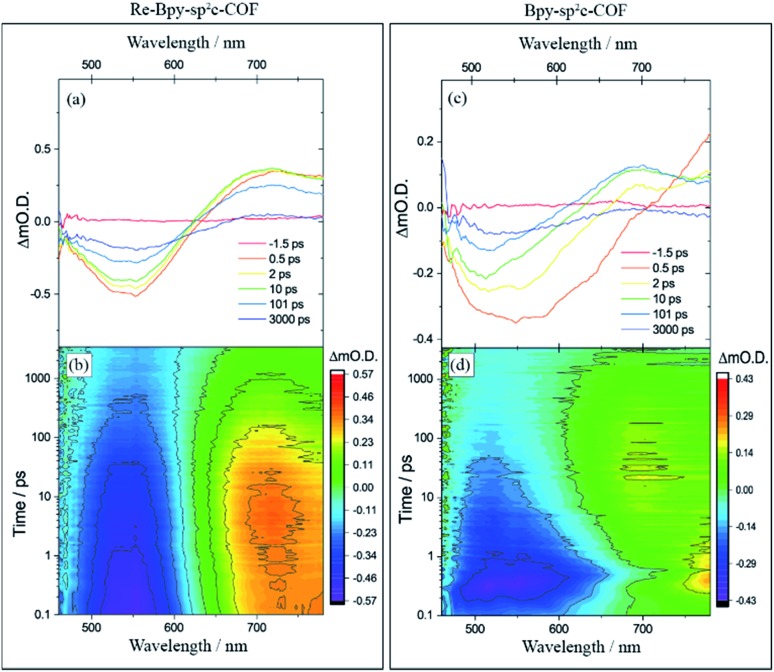
Transient absorption spectra of (a) **Re-Bpy-sp^2^c-COF** and (c) **Bpy-sp^2^c-COF** at pump-probe time delays chosen to highlight the changing nature of the excited electronic states probed, and the complete transient absorption surface probed (b) **Re-Bpy-sp^2^c-COF** and (d) **Bpy-sp^2^c-COF**. All spectra are recorded in CH_3_CN following 400 nm excitation.

To further explore the photophysics of the system we have also carried out transient absorption (TA) spectroscopic studies on both **Re-Bpy-sp^2^c-COF** and **Bpy-sp^2^c-COF** (Fig. 4). Following excitation at 400 nm, 800 μW (5 kHz) of **Bpy-sp^2^c-COF** we observe complex TA spectra with broad negative bands between 450 to *ca.* 700 nm that formed within 0.5 ps. There is minimal absorption by the ground state of **Bpy-sp^2^c-COF** (Fig. 2d) at wavelengths longer than 600 nm. Therefore, the negative signal is proposed to be the overlap of stimulated emission from both the conjugated **Bpy-sp^2^c-COF** structure (*λ*_max_ = 640 nm) and the exfoliated **Bpy-sp^2^c-COF** (*λ*_max_ = 475 nm), overlapped with the ground state bleach, giving rise to the complex shape. The complex nature of the bleach makes determining accurate kinetics challenging, therefore we use *t*_50%_ (the time taken for the bleach to decrease by 50%) as a rough measure of the lifetime of the photogenerated excited state. For **Bpy-sp^2^c-COF** at 550 nm, *t*_50%_ = *ca.* 5 ps (Fig. S46†). Within 0.5 ps a photoinduced absorption (PIA) is present at 770 nm, which decays within 10 ps to form a new PIA centred at 700 nm that decays over the course of the experiment to leave only a small PIA by 3 ns.

**Re-Bpy-sp^2^c-COF** shows a simpler TA spectrum following 400 nm excitation (Fig. 4). A negative band is formed, centred at 540 nm, again assigned to a combination of ground state bleaching and stimulated emission from the exfoliated COF framework (*λ*_max_ = 475 nm; Fig. S23†). It is notable that this negative feature is substantially narrower than that observed for **Bpy-sp^2^c-COF** and for **Re-Bpy-sp^2^c-COF** at 550 nm, *t*_50%_ = *ca.* 200 ps, significantly longer than observed in the absence of the Re. A PIA centred at *ca.* 770 nm is again observed to be formed within 0.5 ps, with a blue shift of this initially formed PIA observed within the first 5 ps, forming a band centred at ∼720 nm. This state continues to decay over the course of the time-delays probed, as the negative band assigned to ground state bleaching and stimulated emission, recovers. Although no direct spectral fingerprint is observed for the formation of the reduced Re centre by TA spectroscopy in the UV/vis spectral region, it is clear from the simplification of the TA spectra, combined with the greatly increased lifetime of the ground state bleach, that the presence of the Re centre within the COF leads to the formation of a long-lived charge separated (non-emissive) state.

Density functional theory (DFT) and time-dependent (TD) DFT calculations—performed on representative molecular models Bpy-sp^2^c(L) and Re-Bpy-sp^2^c(L) of **Bpy-sp^2^c-COF** and **Re-Bpy-sp^2^c-COF**, respectively—show that the electron affinity (EA) and the ionization potential (IP) of both COFs straddle the reduction potential of CO_2_ to CO, as well as the proton reduction potential, and the oxidation potential of TEOA (Fig. S47 and S48†). This provides a thermodynamic explanation for the ability of **Re-Bpy-sp^2^c-COF** to drive CO_2_ reduction to CO, in the presence of the sacrificial agent TEOA. Relative energy levels of the dye and the molecular COF models confirm that it is thermodynamically allowed for excited electrons on the dye to be transferred to **Re-Bpy-sp^2^c-COF** (Fig. S48†), in line with the dye-sensitization effects observed experimentally.

TD-CAM-B3LYP calculations predict that the lowest-energy, excited electronic state (S1) for both Bpy-sp^2^c(L) and Re-Bpy-sp^2^c(L) corresponds to the LUMO ← HOMO transition, with a strong oscillator strength (Table S4†). Electron distributions of the excited-state frontier orbitals show that for both Bpy-sp^2^c(L) and Re-Bpy-sp^2^c(L) the HOMO orbital is predominantly located on the pyrene unit of the COF, with the LUMO orbital mainly located on the bipyridine unit (with or without ligated Re complex; Fig. S49 and S50†). Analyses of excited-state, inter-fragment charge transfer between the building units of the COFs indicate that appreciable amounts of electrons are transferred from the pyrene fragment to the bipyridine fragment (Table S5†), with a sizable electron–hole distance as measured by the charge centroids of the orbitals involved (Δ*r* in Table S4†). Our computational results clearly support that there is electron transfer from the COF backbone to the catalytically active Re complex upon electronic excitation.

The CO_2_ reduction mechanism of the COF compared to the homogenous catalysts is therefore different. Here we propose pyrene excitation to a bipyridine based LUMO. In contrast, in solution excitation upon irradiation forms a metal to bipyridine excited state (^3^MLCT) which is then quenched by an electron donor.^43^

Crystallinity^25^ and accessible surface area^44^ have been shown to be important factors for the photocatalytic activity of organic photocatalysts. To probe whether these factors also influence the performance of the COF in photocatalytic CO_2_ reduction, we synthesized an amorphous analogue by using 1,4-dioxane instead of a 1,2-dichlorobenzene/1-butanol mixture under otherwise exactly the same experimental conditions, Re-Bpy-sp^2^c-P (PXRD, see Fig. S12†), which shows low CO_2_ uptakes and BET surface area (Fig. S19 and S39†). Despite having comparable FT-IR, UV-visible and PL spectra (Fig. S8, S15 and S24†), the amorphous polymer showed significantly lower activity for CO_2_ reduction (Table S2†) after being loaded with Re with a TON of 2.3 after 12 hours compared to 12.9 for **Re-Bpy-sp^2^c-COF**. This highlights that morphological properties, such as crystallinity and porosity, are important in these materials.

We showed previously that COFs that have accessible pores can potentially act as a host for dyes giving rise to increased photocatalytic activity for hydrogen production.^25^ Here, we used (Ir[dF(CF_3_)ppy]_2_(dtbpy))PF_6_ (ppy = 2-phenylpyridine, tbpy = 4,4′-di-*tert*-butyl-2,2′-dipyridyl) in conjunction with **Re-Bpy-sp^2^c-COF** to further enhance the photocatalytic performances. Different amounts of the dye were used, and the CO production rates were enhanced by 32% and 84% compared to the unsensitized COF when using 0.3 mmol and 1.0 mmol of the dye, respectively, with 1 mg COF over 5 hours (Fig. 3e). The H_2_ production rates were unaffected. The highest CO production rates were 1400 μmol h^–1^ g^–1^, with a selectivity of 86% for CO, over 5 hours from **Re-Bpy-sp^2^c-COF** loaded with 1.0 mmol dye. It appears to be an electron transfer mechanism between the dye and the COF *via* oxidative quenching as suggested by emission quenching experiments (Fig. S32†).

Finally, we explored **Re-Bpy-sp^2^c-COF** loaded with additional *in situ* photodeposited colloidal Pt as a photocatalyst for syngas production, hence, simultaneous evolution of CO and H_2_. Syngas is used in chemical industry on large scale for processes, such as Fischer–Tropsch, and control of the ratio is important. The production of syngas with tunable ratio of CO and H_2_ has been reported for electrocatalysts^45^,^46^ and inorganic photocatalysts.^47^,^48^ By adding different amounts of Pt, **Re-Bpy-sp^2^c-COF** could produce high rates of CO-rich or H_2_-rich mixtures ranging from approximately 4 : 1 to 1 : 10 for CO : H_2_ (Fig. 3f).

## Conclusions

In conclusion, we have synthesized a new porous, crystalline bipyridine-containing sp^2^c-COF, which was post-synthetically modified with a rhenium complex to enhance the photocatalytic CO_2_ reduction performance. **Re-Bpy-sp^2^c-COF** achieved a CO production rate of 1040 μmol g^–1^ h^–1^ with 81% selectivity over H_2_ over 17.5 h illumination. This performance was enhanced over 5 hours by up to 84% by dye-sensitization, giving a CO production rate of 1400 μmol h^–1^ g^–1^ and a CO/H_2_ selectivity of 86%. Based on a range of experimental and computational techniques it appears that **Re-Bpy-sp^2^c-COF** operates by a markedly different mechanism compared to the homogeneous catalyst [Re(bpy)(CO)_3_Cl] which the **Re-Bpy-sp^2^c-COF** also outperforms in terms of stability. Crystallinity and porosity seem to be important in these materials since an amorphous, low-porosity analogue showed almost no photocatalytic activity.

## Author contributions

A. I. C. and Z. F. conceived the project. Z. F. synthesized and characterized the materials and performed photocatalysis experiments. L. C. conceived the modelling strategy and L. C. and X. W. performed the calculations. S. Y. C. carried out PXRD analyses. R. S. S. performed the TCSPC experiments. G. N. and A. J. C. carried out isotopic labelling experiments. L. L. performed photoelectrochemical measurements. A. M. G. carried out TA measurements and analysed the results with A. J. C. R. C. and X. L. configured the photocatalysis platform, including setup and methods for GC. R. C., X-Y. W. and L. C. interpreted the gas sorption isotherms. M. B. and X-Y. W. performed STEM imaging. A. I. C., R. S. S. and L. C. co-supervised the project. A. V. provided expertise and feedback. Data was interpreted by all authors and the manuscript was prepared by A. I. C., R. S. S., L. C., and Z. F.

## Conflicts of interest

There are no conflicts to declare.

## Supplementary Material

Supplementary information
