# Supplementary material for: A stable covalent organic framework for photocatalytic carbon dioxide reduction
Source: Chem Sci. 2019 Nov 21;11(2):543–50. doi: 10.1039/c9sc03800k (PMC7069507; doi:10.1039/c9sc03800k)
Supplement: Supplementary file 1 [file SC-011-C9SC03800K-s001.pdf]

Electronic Supplementary Material (ESI) for Chemical Science.
This journal is © The Royal Society of Chemistry 2019

A Stable Covalent Organic Framework for Photocatalytic Carbon Dioxide Reduction

Zhiwei Fu,[1] Xiaoyan Wang,[1] Adrian M. Gardner,[2] Xue Wang,[1,3] Samantha Y. Chong,[1] Gaia Neri,[2] Alexander J. Cowan,[2] Lunjie Liu,[1] Xiaobo Li,[1] Anastasia Vogel,[1] Rob Clowes,[1] Matthew Bilton,[4] Linjiang Chen,[1,3*] Reiner Sebastian Sprick,[1*] and Andrew I. Cooper[1,3*]

1 Department of Chemistry and Materials Innovation Factory, University of Liverpool, 51 Oxford Street, Liverpool, L7 3NY, U.K.

2 Stephenson Institute for Renewable Energy, University of Liverpool, Chadwick Building, Peach Street, Liverpool, L69 7ZF, U.K.

3 Leverhulme Research Centre for Functional Materials Design, Materials Innovation Factory and Department of Chemistry, University of Liverpool, Oxford Street, Liverpool L7 3NY, U.K.

4 Imaging Centre at Liverpool, University of Liverpool, Liverpool L69 3GL, U.K.

*Corresponding author. Emails: aicooper@liverpool.ac.uk, ssprick@liverpool.ac.uk, lchen@liverpool.ac.uk

Table of Contents

1. Materials and methods 4
   1.3 Thermogravimetric analysis 4
   1.4 Gas sorption analysis 4
   1.5 Fourier-transform infrared spectroscopy 4
   1.6 UV-Visible absorption spectra 4
   1.8 Scanning transmission electron microscopy 5
   1.9 Isotopic labelling experiments 5
   1.10 Photoelectrochemical measurements 5
   1.11 Inductively coupled plasma - optical emission spectrometry (ICP-OES) analysis 5
   1.12 Transmission and backscattering experiments 6
   1.13 Time-correlated single photon counting (TCSPC) measurements 6
   1.14 Determination of apparent quantum yield (AQY) for CO production 6
   1.15 Photocatalytic CO_2 reduction experiments 6
   1.16 X-ray photoelectron spectroscopy (XPS) measurements 7
   1.17 Transient absorption (TA) spectroscopic 7
2. Synthetic procedures 8
3. NMR Spectra 10
4. Fourier-transform infrared spectroscopy 12
5. Powder X-ray diffraction 14
6. UV-visible spectra 17
7. Photocatalytic CO_2 reduction experiments 19
8. Scanning electron microscopy 20
9. Thermogravimetric analysis 21
10. CO_2 uptake 22
11. Time-correlated single photon counting experiments 23
12. Energy-dispersive X-ray spectroscopy 33
13. Wavelength-dependent CO_2 reduction experiments 34
14. Transmission and backscattering experiments 35
15. Gas sorption isotherms 36
16. X-Ray photoelectron spectroscopy 37
17. Photoelectrochemical measurements 38
18. Control experiments and previously reported data 42
19. Density functional theory (DFT) and time-dependent DFT (TD-DFT) calculations 44

1. Materials and methods

All reagents were obtained from Sigma-Aldrich, TCI Europe or Fluorochem Ltd. Anhydrous solvents were purchased from Acros Organics or Fisher Scientific. All chemicals were used as received and without further purification. 5,5'-Bis(cyanomethyl)-2,2'-bipyridine and 1,3,6,8-tetrakis(4-formylphenyl)pyrene (TFPPy) were synthesized based on previous literature procedures.[1,2]

1.1 Solution nuclear magnetic resonance

^{1}H and $^{13}C\{^{1}H\}$ NMR spectra were recorded on a Bruker Avance 400 NMR spectrometer, operating at frequencies of 400 MHz and 100 MHz, respectively.

1.2 Powder X-ray diffraction

Powder X-ray diffraction (PXRD) measurements were carried out on a Panalytical Empyrean diffractometer, equipped with a Cu X-ray source (λ = 1.5418 Å, Cu Kα), PIXcel3D detector and X-ray focusing mirror. The loose powdered sample was held on Mylar film in aluminium well plates and screened in high throughput transmission mode. Powder diffraction analysis was performed using TOPAS-Academic.[3]

1.3 Thermogravimetric analysis

Thermogravimetric analysis (TGA) was measured on an EXSTAR6000 with an automated vertical overhead thermobalance under nitrogen flow, ramping heating at 10 °C min^{-1} from 25 °C to 600 °C.

1.4 Gas sorption analysis

Nitrogen adsorption and desorption were measured at 77.3 K using a Micromeritics ASAP 2020 volumetric adsorption analyzer. Powder samples were degassed offline at 393 K for 12 hours under dynamic vacuum (10^{-5} bar) before analysis. BET surface areas were fitted over relative pressure (p/p°) from 0.1 to 0.2. Pore size distributions of COFs were obtained from the adsorption data by fitting a nonlocal density functional theory (NL-DFT) model with method of N_2-cylindrical pores-oxide surface. CO_2 isotherms were collected up to a pressure of 1200 mbar on a Micromeritics ASAP 2020 at 273 K and 298 K.

1.5 Fourier-transform infrared spectroscopy

FT-IR spectra were collected on a Bruker Tensor 27 FT-IR spectrometer. Samples were prepared as KBr disks before analyzing for 16 scans with a resolution of 4 cm^{-1}.

1.6 UV-Visible absorption spectra

UV-Visible absorption spectra were measured on an Agilent Cary 5000 UV-Vis-NIR Spectrometer by measuring the reflectance of powders in the solid-state.

1.7 Scanning electron microscopy

The morphology of the materials was studied using a Hitachi S-4800 cold field emission scanning electron microscope (FE-SEM). Samples were prepared by depositing the powders with an adhesive high-purity carbon tab on Hitachi M4 aluminium stubs.

1.8 Scanning transmission electron microscopy

STEM and EDX images were obtained on a JEOL 2100F Cs-corrected analytical FEG S/TEM operating at 200 kV, and fitted with an EDAX Octane T Optima windowless 60 mm^2 SDD EDX detector. The samples were prepared by drop-casting sonicated ethanol suspensions of the materials onto a copper grid.

1.9 Isotopic labelling experiments

$^{13}CO_2$ Labelling experiments were carried on a Bruker Vertex 70V Fourier-transform infrared spectrometer with an argon-purged custom-made gas IR cell. A vial containing COF powders, acetonitrile and triethanolamine was purged with $^{13}CO_2$ (Sigma-Aldrich, 99 atom % ^{13}C, <3 atom % ^{18}O) for 3 minutes, then it was illuminated for 4 hours using a 300 W Xe light source equipped with $\lambda > 420$ nm filter. Gas from the headspace of the vial (500 µL) was injected into the gas IR cell and a spectrum was measured (32 scans with a resolution of 0.5 cm^{-1}).

1.10 Photoelectrochemical measurements

1 mg of the photocatalyst was dispersed in 0.1 mL acetonitrile and ultrassonicated for 10 minutes giving a homogenous suspension. Fluoride-tin oxide (FTO) glass slides were covered with a copper mask giving an area of 0.28 cm^2. 10 µL of the suspension was drop-casted on the FTO glass and dried overnight at room temperature. The photocurrent response was measured using a three-electrode setup with a working electrode (COF on FTO glass), counter electrode (Pt wire), and reference electrode (Ag/AgCl). An Oriel Instruments LSH-7320 Solar Simulator (IEC ABA certified) with 1 Sun output was used to illuminate the sample. A 0.1 M Na_2SO_4 (pH = 7) solution with a bias voltage of -0.1 V was used for the measurement. The EIS spectra were recorded by applying a 10 mV AC signal in the frequency range from 100 kHz to 0.01 Hz. The potential converted to reversible hydrogen electrode (RHE) using the following equation[4]:

$$E_{RHE} = E_{Ag/AgCl} + 0.059\text{pH} + E^0_{Ag/AgCl} (E^0_{Ag/AgCl} = 0.199\text{V})$$

1.11 Inductively coupled plasma - optical emission spectrometry (ICP-OES) analysis

Samples were digested in nitric acid (67–69%, trace metal analysis grade) with a microwave using an in-house procedure. The obtained solutions were diluted with water before the measurement by Spectro Ciros ICP-OES and the instrument was calibrated with standards in aqueous solution.

1.12 Transmission and backscattering experiments

Samples were tested on a Formulaction S.A.S. Turbiscan AGS with an 880 nm NIR diode and a detector at 180° or 45° (relative to the light source) in a cylindrical glass cell. Before the measurements, samples were dispersed in 20 mL acetonitrile and sonicated for 15 minutes. Then, the transmission and backscattering of the suspensions were measured in cylindrical glass cells from 5000 to 30,000 μm every 40 μm.

1.13 Time-correlated single photon counting (TCSPC) measurements

TCSPC experiments were performed on an Edinburgh Instruments LS980-D2S2-STM spectrometer equipped with picosecond pulsed LED excitation sources and a R928 detector, with a stop count rate below 3%. An EPL-375 diode (λ = 370.5 nm, instrument response 100 ps, fwhm) with a 450 nm high pass filter for emission detection was used. Suspensions were prepared by ultrasonicating the materials in acetonitrile or acetonitrile with triethanolamine (30/1) purged with N_2 and CO_2. The instrument response was measured with colloidal silica (LUDOX® HS-40, Sigma-Aldrich) at the excitation wavelength without filter. Decay times were fitted in the FAST software using suggested lifetime estimates.

1.14 Determination of apparent quantum yield (AQY) for CO production

The apparent quantum yield of CO production was determined using monochromatic LED light (λ = 420 nm). The reactions were conducted on the same photochemical experimental setup under the optimized reaction conditions. For the experiments, COFs (1 mg) was suspended in acetonitrile and triethanolamine (30:1 vol. mixture, 5 mL). The illuminated area was 8 cm^2 and the light intensity was measured by a ThorLabs PM100D Power and Energy Meter Console with a ThorLabs S120VC photodiode power sensor. The AQY was calculated as follow:

$$AQY\% = 2 \times [(n\ CO) \times N_A \times \hbar \times c)] \times 100\% / (I \times S \times t \times \lambda)$$

Where, N_A is Avogadro constant (6.022×10^{23} mol^{-1}), ħ is the Planck constant (6.626×10^{-34} J s), c is the speed of light (3×10^8 m s^{-1}), S is the irradiation area (cm^2), I is the intensity of irradiation light (W cm^{-2}), t is the photoreaction time (s), λ is the wavelength of the monochromatic light (m).

1.15 Photocatalytic CO_2 reduction experiments

A quartz flask was charged with the COF powder (1 mg), acetonitrile (MeCN) and triethanolamine (TEOA) (30:1 vol. mixture, 5 mL) and sealed with a septum. The resulting suspension was ultrasonicated for 5 minutes and then purged with CO_2 for 15 minutes. The reaction mixture was illuminated with a 300 W Newport Xe light source (model: 6258, Ozone free) equipped with a λ > 420 nm cut-off filter. Gaseous products were taken with a gas-tight syringe and run on a Shimadzu GC-2014 gas chromatograph equipped with a ShinCarbon ST micropacked column (Restek 80-100 mesh, 2 m length, 0.53 mm inner diameter) and a thermal conductivity detector.

1.16 X-ray photoelectron spectroscopy (XPS) measurements

X-ray photoelectron spectroscopy (XPS) measurements were performed using a Kratos AXIS Ultra DLD instrument. The chamber pressure during the measurements was 5×10^{-9} Torr. Wide energy range survey scans were collected at pass energy of 80 eV in hybrid slot lens mode and a step size of 0.5 eV. Wide scan and high-resolution data on the C 1s, O 1s, Cl 2p, Re 4f and Co 2p photoelectron peaks was collected at pass energy 20 eV over energy ranges suitable for each peak, and collection times of 5 min, step sizes of 0.1 eV. The charge neutraliser filament was used to prevent the sample charging over the irradiated area. The X-ray source was a monochromated Al Kα emission, run at 10 mA and 12 kV (120 W). The energy range for each 'pass energy' (resolution) was calibrated using the Kratos Cu 2p3/2, Ag $3d_{5/2}$ and Au $4f_{7/2}$ three-point calibration method. The transmission function was calibrated using a clean gold sample method for all lens modes and the Kratos transmission generator software within Vision II.

1.17 Transient absorption (TA) spectroscopic

The apparatus employed to obtain transient absorption, TA, spectra of the COFs of interest consists of an Ytterbium laser system (PHAROS Short-Pulse 10 W, PH1-SP-10W, Light Conversion) with an output power of 10 W, wavelength of 1028 nm, repetition rate of 10 kHz and pulse duration of ~180 fs. Of this, ~1 W is used to drive an Optical Parametric Amplifier, OPA (ORPHEUS, Light Conversion) in tandem with a second harmonic generation module (LYRA, Light Conversion) in order to generate radiation centred at 400 nm with a bandwidth (FWHM) of 3 nm.

This 400 nm output was used as the pump source for subsequent TA measurements which employed a commercial TA spectrometer (HARPIA, Light Conversion). The probe light was visible white light super continuum generated by focusing < 0.1 W of 1028 nm radiation onto a sapphire window. Variable delay times between the pump and probe beams were obtained by passing the pump beam through a multi-pass mechanical delay stage allowing pump-probe delays up to 3.6 ns to be achieved. The pump and probe beams were focused to 600 and 400 μm spots at the sample. The pump laser beam was chopped, allowing, or blocking several pump pulses, resulting in an effective pumping repetition rate of 5 kHz to be obtained; the state of pumping of the sample (i.e. pumped/unpumped), along with stability of the pump laser power, is monitored using a photodiode. The power of the chopped beam incident on the sample was 0.8 mW. The samples were suspensions of the COF of interest, in either pure acetonitrile, or a 30:1 mixture of acetonitrile and TEOA, held within a quartz cuvette with a 2 mm path length.

The white light supercontinuum was collimated and routed to the detector. Here the white light was spectrally dispersed by a spectrograph (Kymera 193i, Andor), employing a grating of 150 lines/mm, blazed at 800 nm, and detected using an NMOS detector (S3901, Hamamatsu).

2. Synthetic procedures

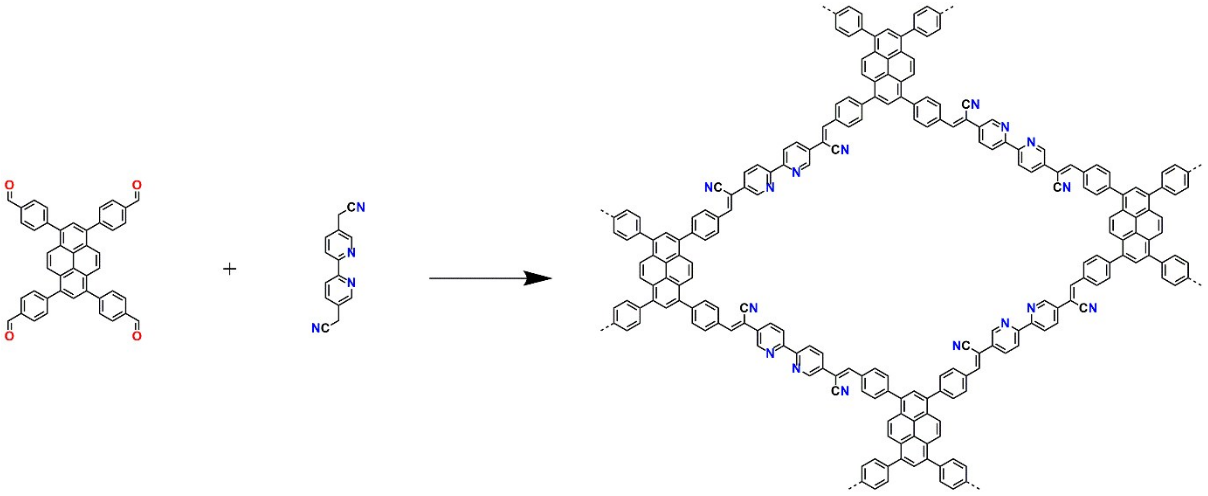

Figure S1. Scheme of the synthesis of Bpy-sp^{2}c-COF.

Synthesis of Bpy-sp^{2}c-COF: A Pyrex tube (10 mL) was charged with TFPPy (14.8 mg, 0.024 mmol) and 5,5'-bis(cyanomethyl)-2,2'-bipyridine (11.5 mg, 0.048 mmol), 1,2-dichlorobenze (0.5 mL), 1-butanol (0.5 mL) and aqueous KOH solution (0.1 mL, 4 M). The mixture was ultrasonicated for two minutes and then flash frozen at 77 K (liquid N_2 bath) and degassed through three freeze-pump-thaw cycles and sealed under vacuum using a Schlenk line and oil pump. The tube was heated at 120 °C for 3 days. After cooling to room temperature, the precipitated was washed with HCl (aq. 1 M), water, THF and methanol three times, respectively. The resulting powder was subjected to Soxhlet extraction with THF for two days. The powder was collected and dried at 120 °C under vacuum overnight to afford yellow crystallites in 82% yield. Anal. Calcd for $(C_{72}H_{42}N_8)_n$: C, 84.85; H, 4.15; N, 10.99. Found: C, 70.09; H, 4.09; N, 6.99. Note: The yields and microanalysis data were calculated for an infinite structure and also ignoring the presence of end-groups whose nature is unclear. The predicted theoretical surface areas of Bpy-sp^{2}c-COF is 2041.21 m^2 g^{-1}.

Synthesis of Bpy-sp^{2}c-P: A flask (250 mL) was charged with TFPPy (148 mg, 0.24 mmol) and 5,5'-bis(cyanomethyl)-2,2'-bipyridine (115 mg, 0.48 mmol), 1,4-dioxane (100 mL) and aqueous KOH solution (8 mL, 4 M). The mixture was ultrasonicated for two minutes and then heated at 120 °C for 3 days. After cooling to room temperature, the precipitated was washed with HCl (aq. 1 M), water, THF and methanol for three times, respectively. The resulting powder was subjected to Soxhlet extraction with THF for two days. The powder was collected and dried at 120 °C under vacuum overnight to afford yellow crystallites in 81.50% isolated yield. Anal. Calcd for $(C_{72}H_{42}N_8)_n$: C,84.85; H, 4.15; N, 10.99. C, 70.05; H, 3.52; N, 6.34. Note: The yields and microanalysis data were calculated for an infinite structure and also ignoring the presence of end-groups whose nature is unclear.

Synthesis of Re-Bpy-sp^{2}c-COF and Re-Bpy-sp^{2}c-P: Re-Bpy-sp^{2}c-COF and Re-Bpy-sp^{2}c-P were prepared according a modified literature method.[5] COF or amorphous materials (10 mg) and

$[Re(CO)_5Cl]$ (10 mg, 0.028 mmol) were dispersed and refluxed in 10 mL toluene for 40 min. The dark red product was filtered and washed with methanol for 3 times. The resulting powders was dried under vacuum overnight. ICP-OES analysis shows a Re content in Re-Bpy-sp^{2}c-COF of 18.1 wt. % (0.97 mmol g^{-1}) and Re-Bpy-sp^{2}c-P of 9.4 wt. %. The predicted theoretical surface areas of Re-Bpy-sp^{2}c-COF is 1835.57 m^2 g^{-1}.

Synthesis of $Re(bpy)(CO)_3Cl$: $Re(bpy)(CO)_3Cl$ was prepared according a modified literature method.[6] In a 50 mL round-bottom flask, $Re(CO)_5Cl$ (0.251g, 0.68 mmol), 2,2′-bipyridine (0.106g, 0.68 mmol), and toluene (20 mL) were added together, and the resultant reaction mixture was refluxed under N_2 for 1 h. Upon cooling, the product was vacuum filtered, and rinsed with cold toluene. ^1H NMR (δ, 400 MHz, DMSO-d_6): 9.02 (d, 1H), 8.77 (d, 1H), 8.34(t, 1H), 7.76(t, 1H).

3. NMR Spectra

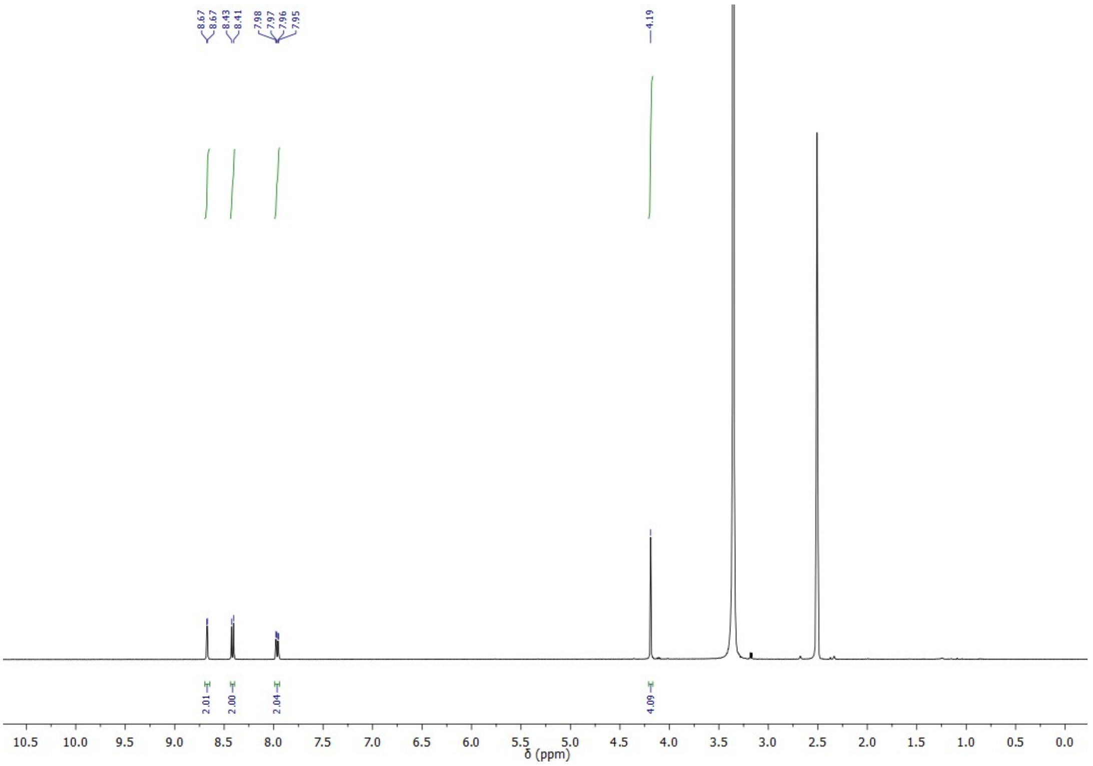

Figure S2. ^{1}H NMR spectrum of 5,5’-bis(cyanomethyl)-2,2’-bipyridine in DMSO-d_6.

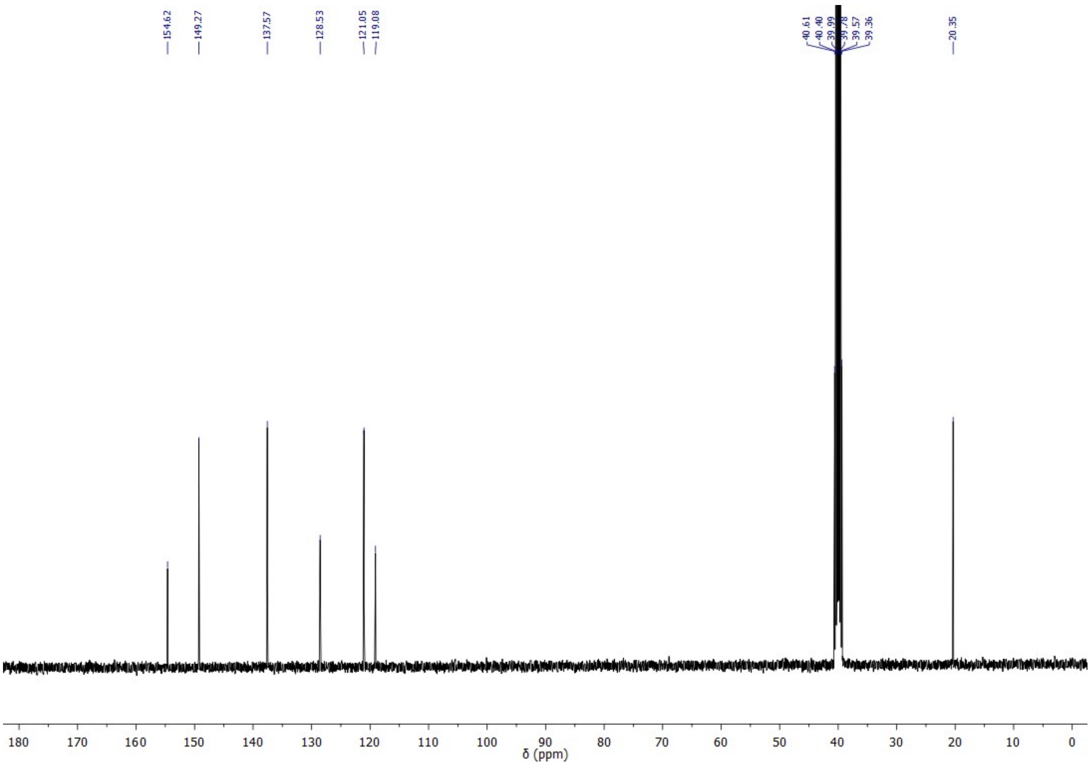

Figure S3. $^{13}C\{^{1}H\}$ NMR spectrum of 5,5’-bis(cyanomethyl)-2,2’-bipyridine in DMSO-d_6.

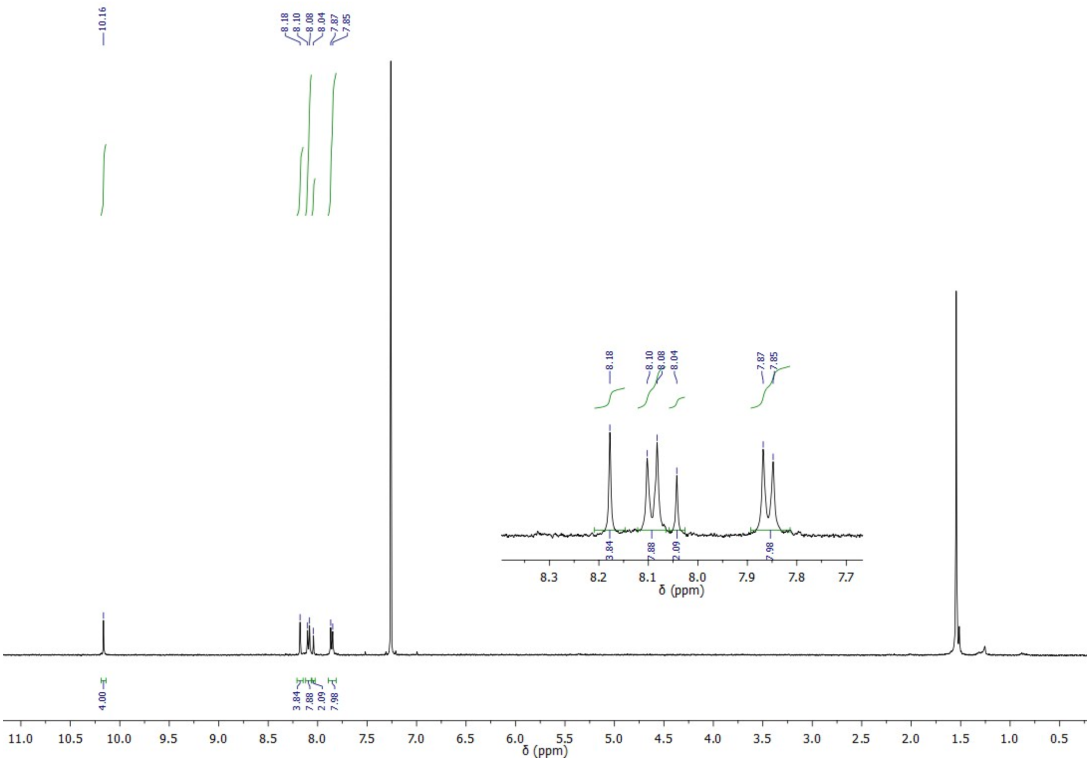

Figure S4. ^{1}H NMR spectrum of 1,3,6,8-tetrakis(4-formylphenyl)pyrene $CDCl_3$.

4. Fourier-transform infrared spectroscopy

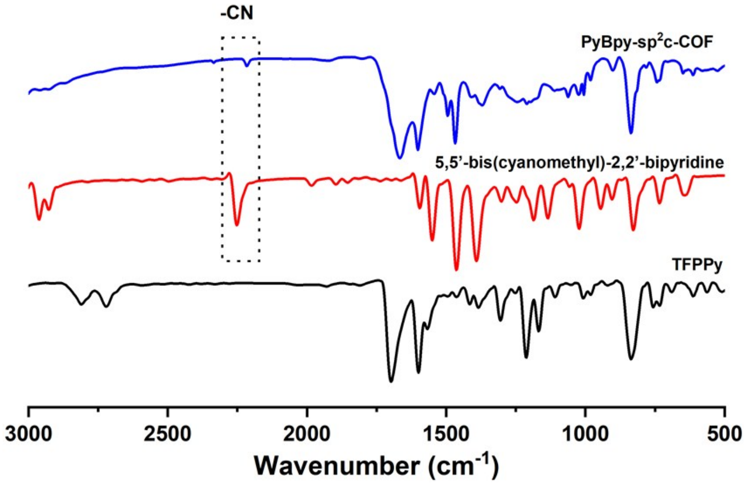

Figure S5. FT-IR spectra of 5,5'-bis(cyanomethyl)-2,2'-bipyridine, TFPPy and **Bpy-sp²c-COF**.

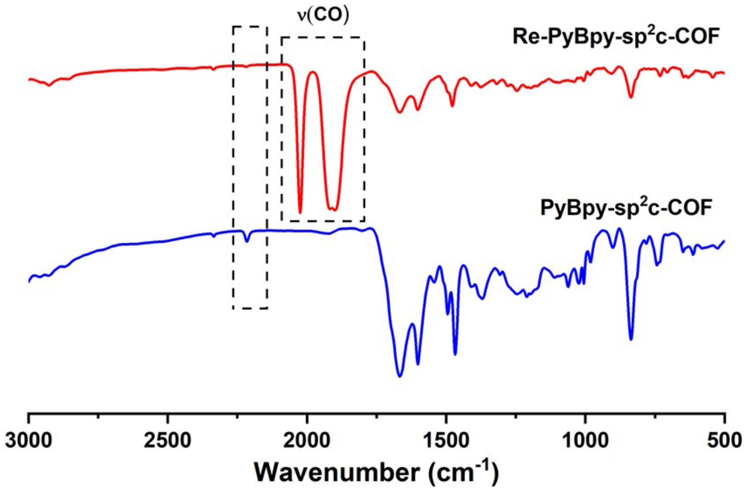

Figure S6. FT-IR spectra of **Bpy-sp²c-COF** and after loading with [Re(CO)$_5$Cl] (**Re-Bpy-sp²c-COF**).

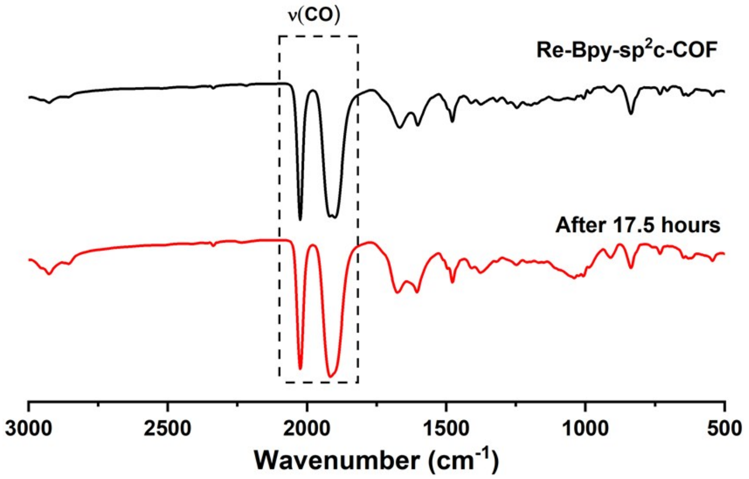

Figure S7. FT-IR spectra of **Re-Bpy-sp^{2}c-COF** before and after 17.5 hours of visible light irradiation (300 W Xe light source, λ > 420 nm).

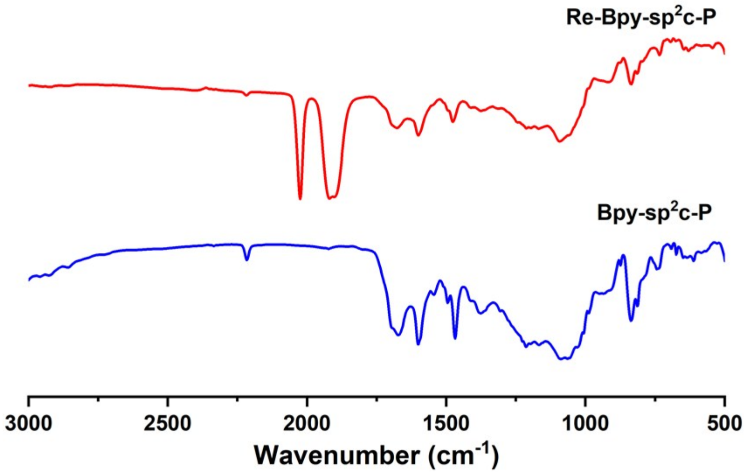

Figure S8. FT-IR spectra of **Bpy-sp^{2}c-P** and after loading with [Re(CO)$_5$Cl] (**Re-Bpy-sp^{2}c-P**).

5. Powder X-ray diffraction

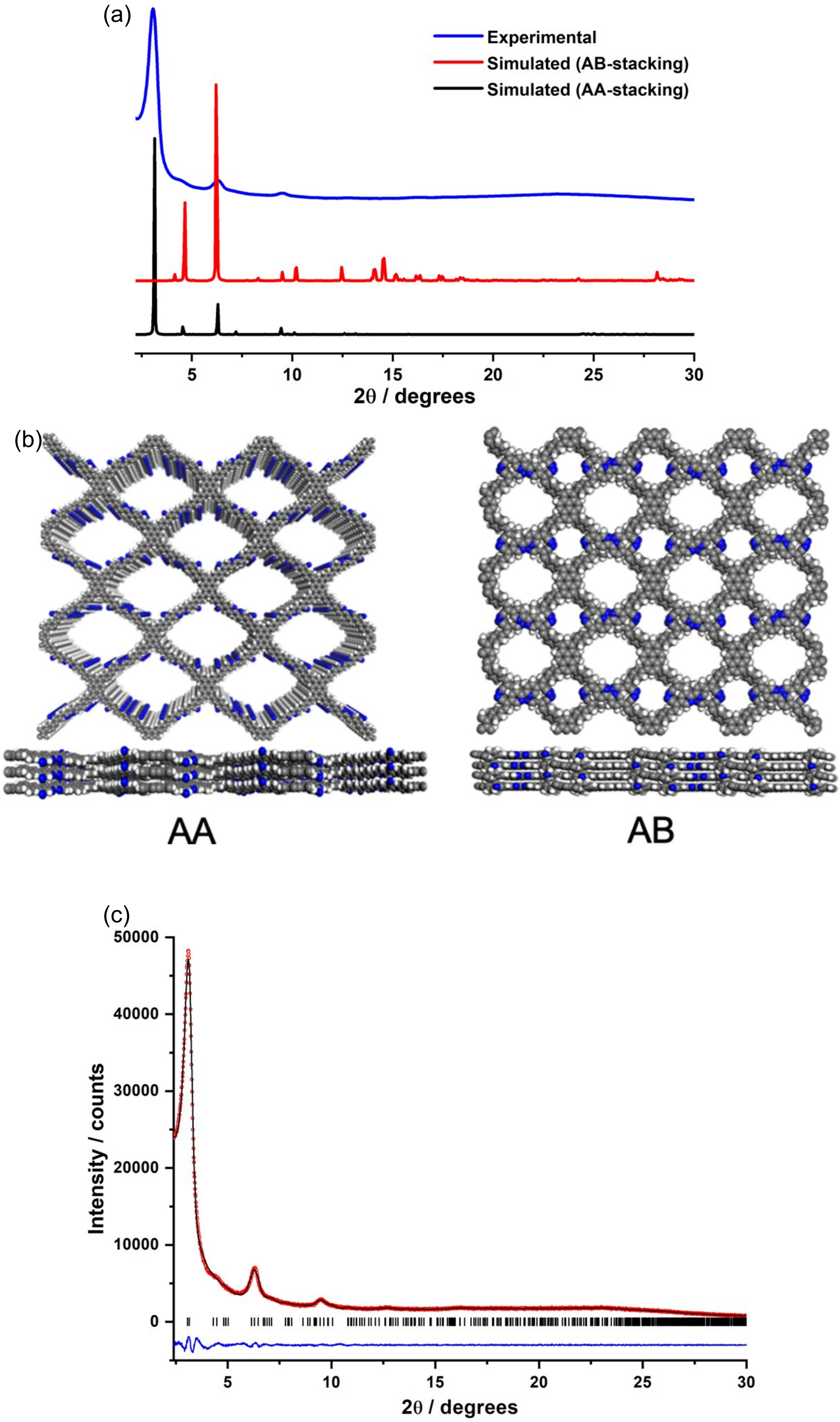

Figure S9. (a) Experimentally observed powder X-ray diffraction pattern of **Bpy-sp²c-COF** (blue) and simulated profiles for AA and AB stacking modes (red and black); (b) Structural models for **Bpy-sp²c-COF** with ideal eclipsed AA and AB stacking patterns, shown parallel to the pore channel along the crystallographic c axis (top) and parallel to the hexagonal layers (bottom); (c) final observed (red circles), calculated (black line) and difference profiles for Le Bail refinement (R_{wp} = 2.55 %, R_p = 1.98 %, χ^2 = 1.49) for **Bpy-sp²c-COF** (a = 39.45(2), b = 40.97(2), c = 3.684(2) Å, α = 91.42(4)°, β = 89.5(2), γ = 90.5(2), V = 5953(5) Å^3, triclinic). Reflection positions are indicated by tick marks.

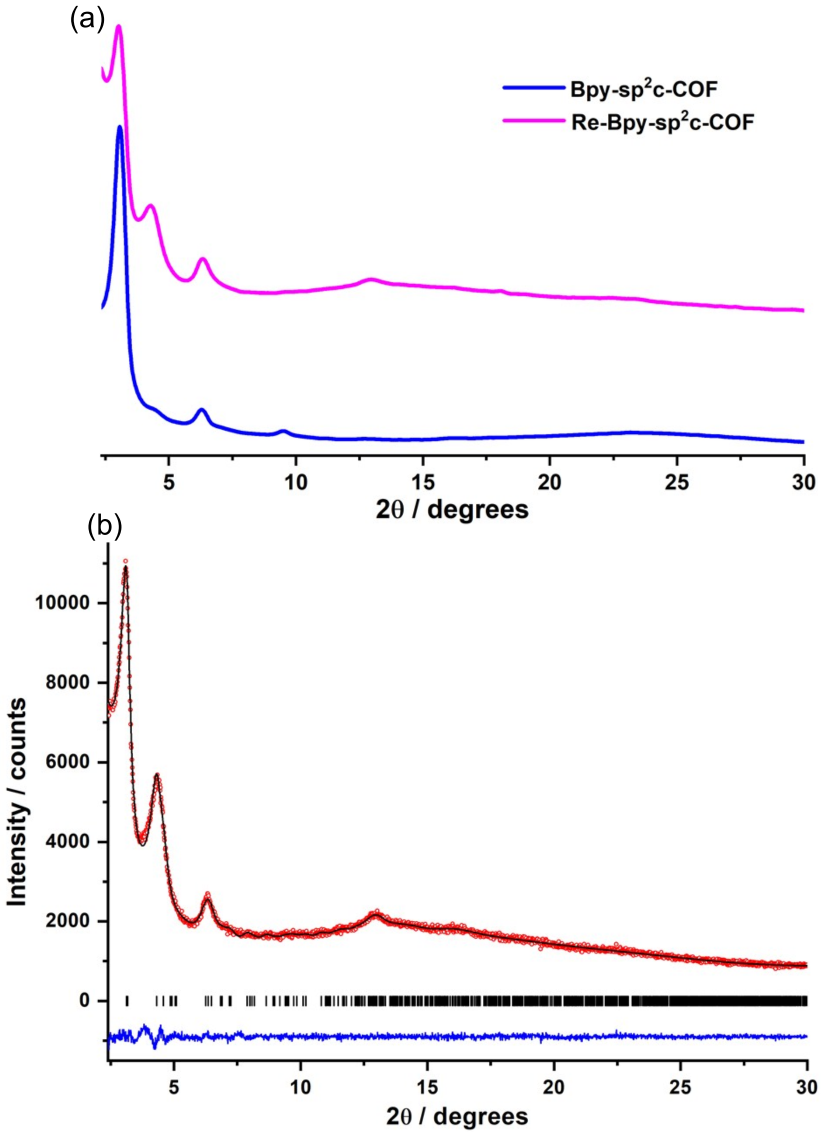

Figure S10. (a) Experimentally observed powder X-ray diffraction pattern of **Bpy-sp²c-COF** (blue), **Re-Bpy-sp²c-COF** (pink); (b) final observed (red circles), calculated (black line) and difference profiles for Le Bail refinement (R_{wp} = 2.49 %, R_p = 1.93 %, χ^2 = 1.24) for **Re-Bpy-sp²c-COF** (a = 38.52(2), b = 40.91(2), c = 7.37(1) Å, α= 91.3(8)°, β= 91.6(5)°, γ= 89.18(5)°, V = 11608(23) Å^3, triclinic). Reflection positions are indicated by tick marks.

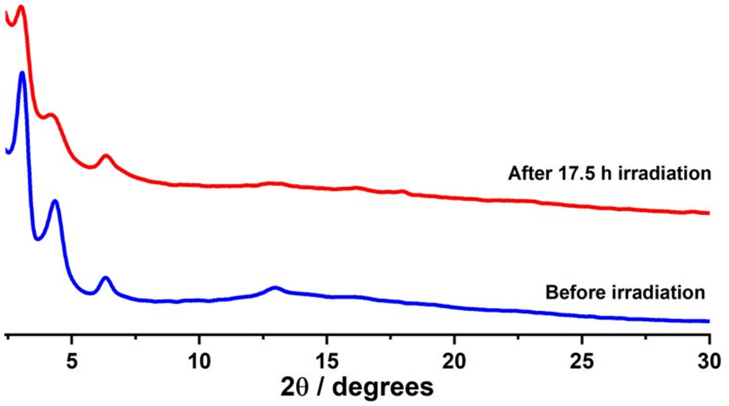

Figure S11. Experimentally observed powder X-ray diffraction pattern of **Re-Bpy-sp²c-COF** before and after 17.5 hours irradiation.

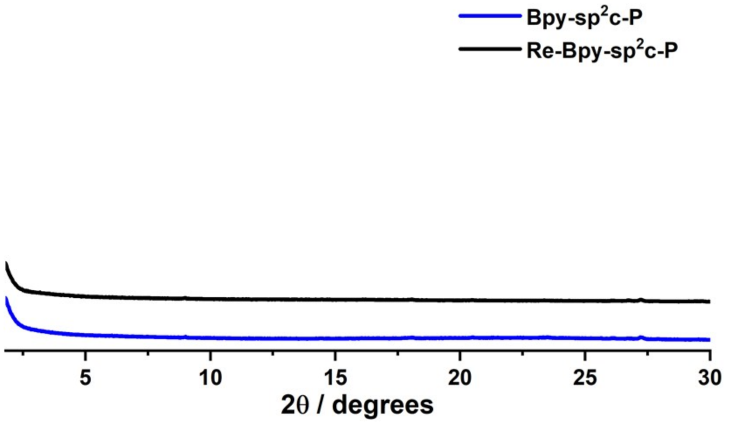

Figure S12. Experimentally observed powder X-ray diffraction pattern of **Bpy-sp²c-P** (blue) and **Re-Bpy-sp²c-P** (black).

6. UV-visible spectra

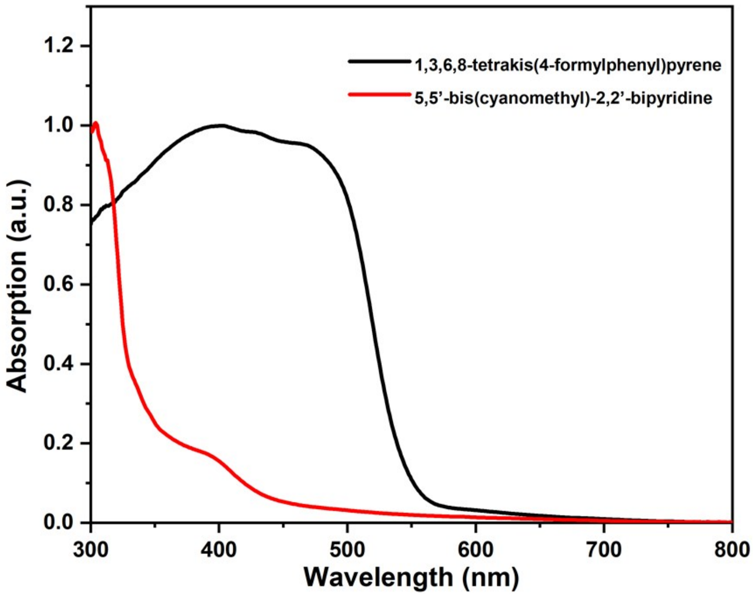

Figure S13. UV-Vis absorption spectra of 5,5'-bis(cyanomethyl)-2,2'-bipyridine and 1,3,6,8-tetrakis(4-formylphenyl)pyrene in the solid-state.

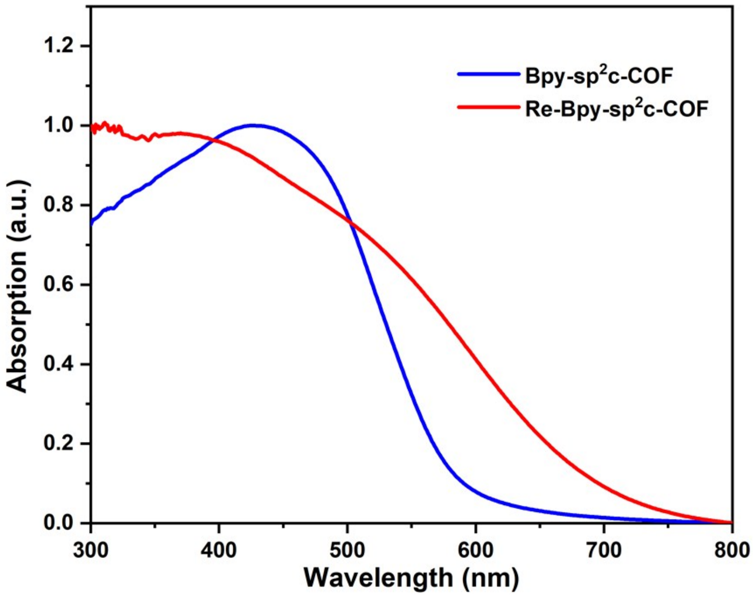

Figure S14. UV-Vis absorption spectra of **Bpy-sp^{2}c-COF** and after loading with [Re(CO)$_5$Cl] (**Re-Bpy-sp^{2}c-COF**) measured in the solid-state.

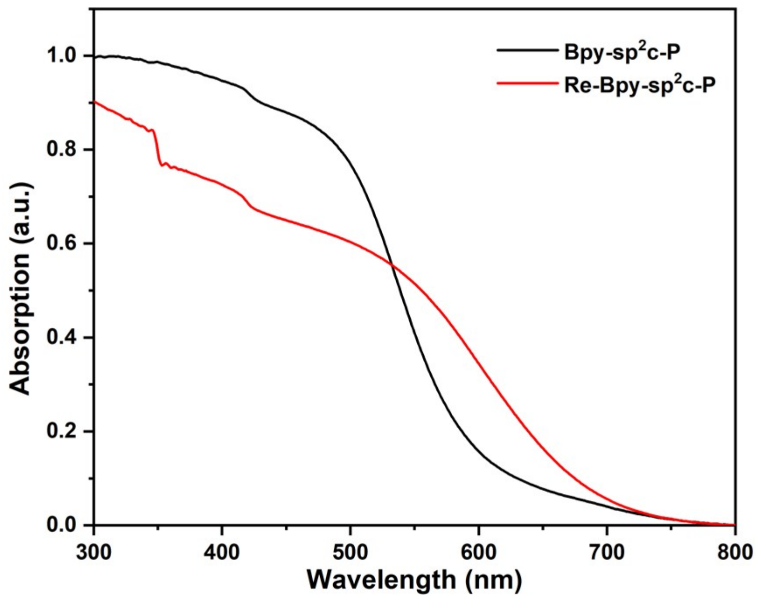

Figure S15. UV-Vis absorption spectra of **Bpy-sp²c-P** and after loading with [Re(CO)$_5$Cl] (**Re-Bpy-sp²c-P**) measured in the solid-state.

7. Photocatalytic CO_2 reduction experiments

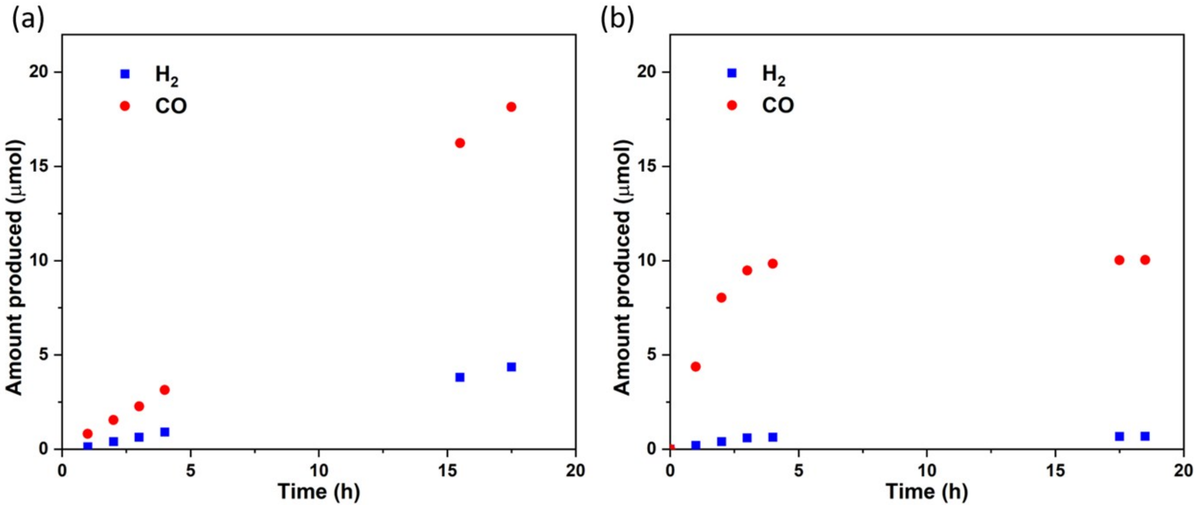

Figure S16. CO_2 reduction experiments of **Re-Bpy-sp²c-COF** (1 mg) (a) and Re(bpy)$(CO)_3$Cl (0.45 mg, 0.97 μmol) (b) from MeCN and TEOA (5 mL, 30/1) under visible light irradiation (λ > 420 nm, 300 W Xe light source). The TONs are 18.7 and 10.3 for Re-Bpy-sp²c-COF and Re(bpy)$(CO)_3$Cl, respectively.

8. Scanning electron microscopy

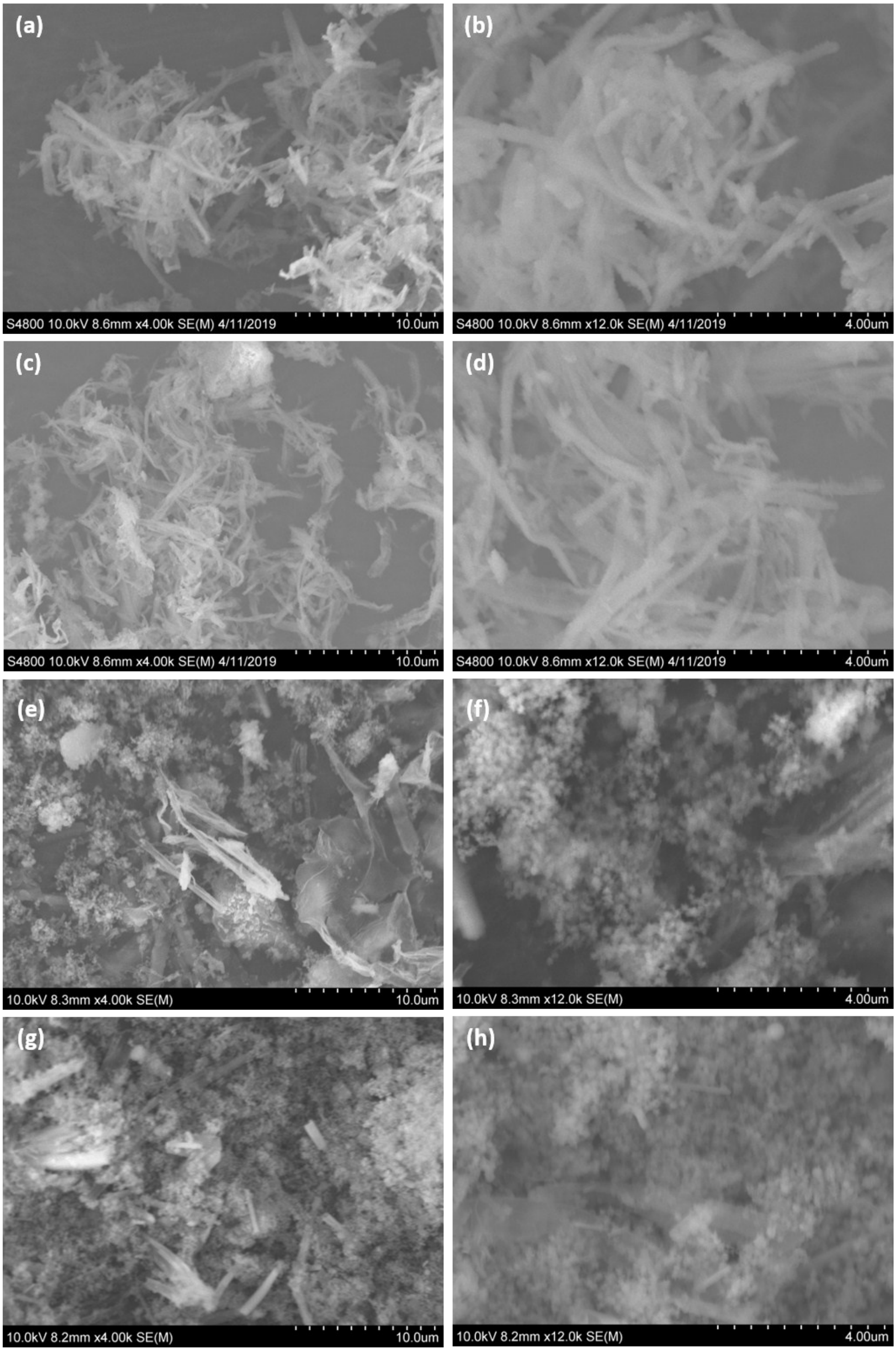

Figure S17. SEM images of **Bpy-sp²c-COF** (a, b), **Re-Bpy-sp²c-COF** (c, d), **Bpy-sp²c-P** (e, f) and **Re-Bpy-sp²c-P** (g, h).

9. Thermogravimetric analysis

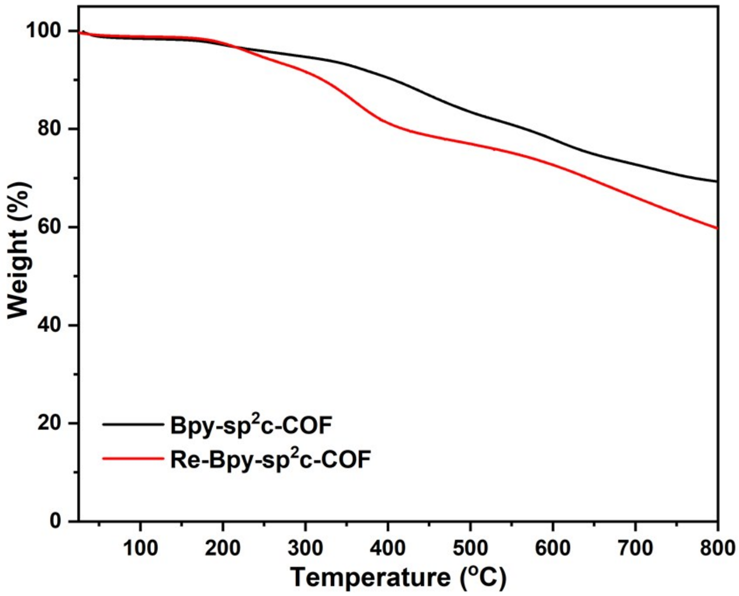

Figure S18. TGA traces of **Bpy-sp²c-COF** and after loading with [Re(CO)$_5$Cl] (**Re-Bpy-sp²c-COF**) measured under nitrogen.

10. CO_2 uptake

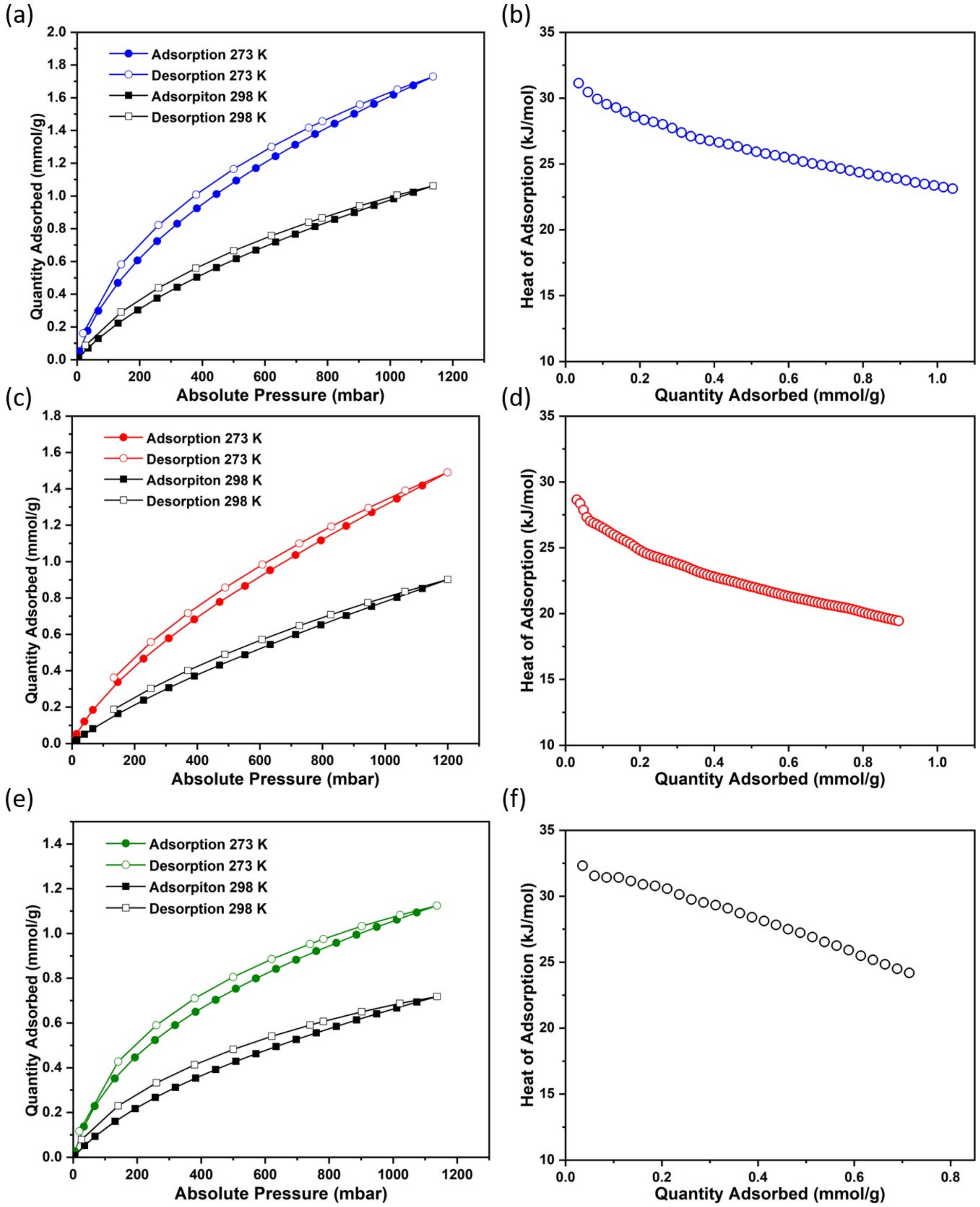

Figure S19. CO_2 sorption isotherms and CO_2 isosteric heat of adsorption calculated from 273 K and 298 K of **Re-Bpy-sp^{2}c-COF** (a, b), **Bpy-sp^{2}c-COF** (c, d) and **Bpy-sp^{2}c-P** (e, f).

11. Time-correlated single photon counting experiments

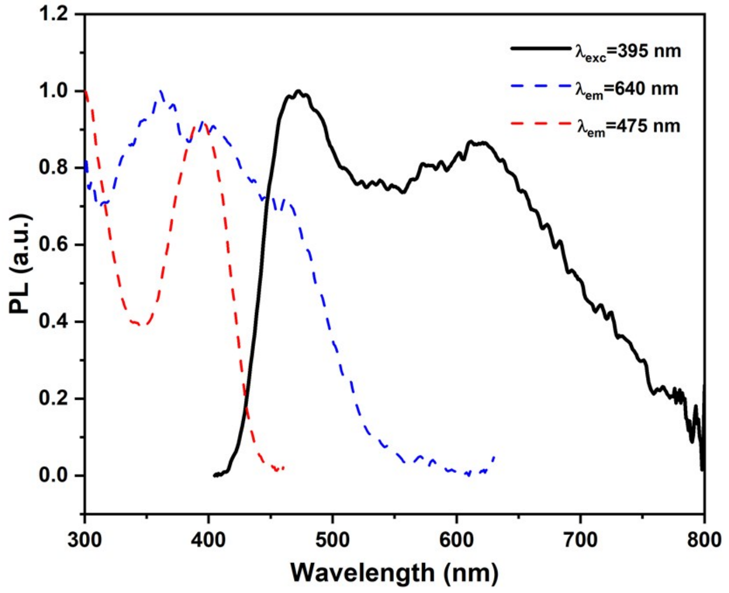

Figure S20. Fluorescence emission and excitation spectra of **Bpy-sp^{2}c-COF** in acetonitrile.

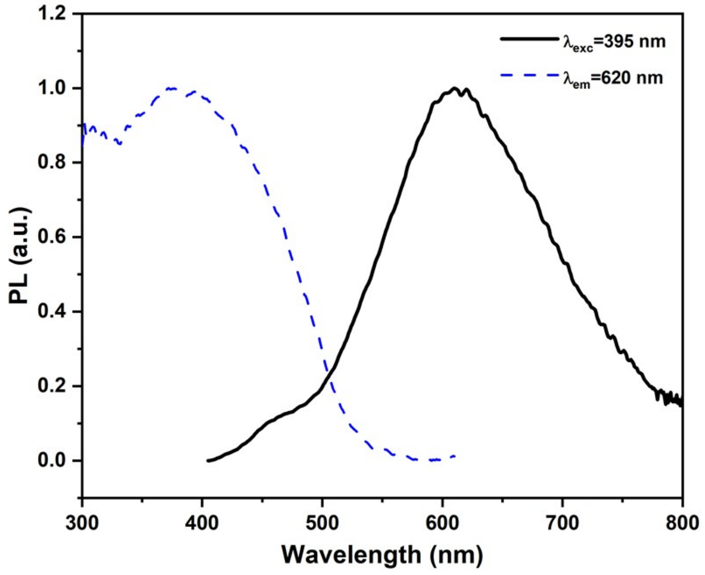

Figure S21. Fluorescence emission and excitation spectra of **Bpy-sp^{2}c-COF** in acetonitrile and triethanolamine (30/1) mixture.

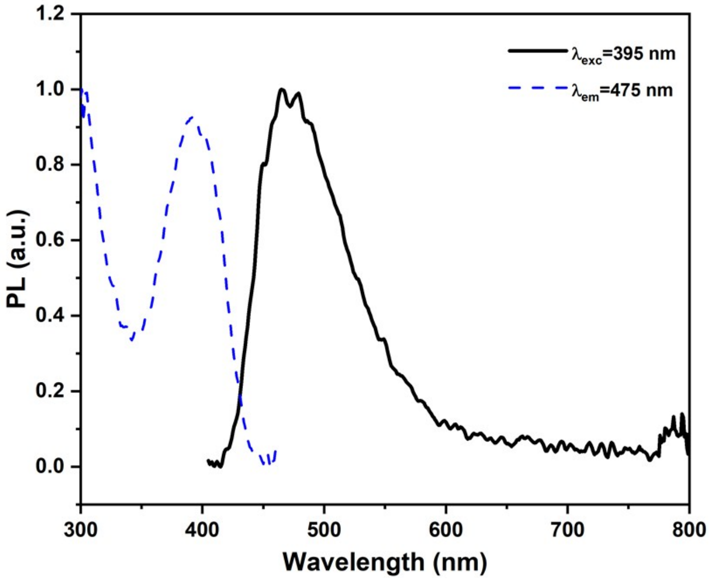

Figure S22. Fluorescence emission and excitation spectra of **Re-Bpy-sp^{2}c-COF** in acetonitrile.

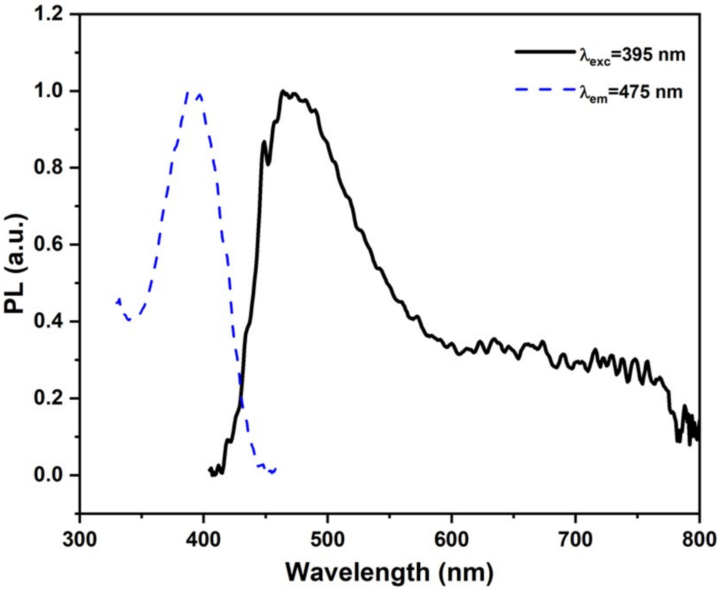

Figure S23. Fluorescence emission and excitation spectra of **Re-Bpy-sp^{2}c-COF** in acetonitrile and triethanolamine (30/1) mixture.

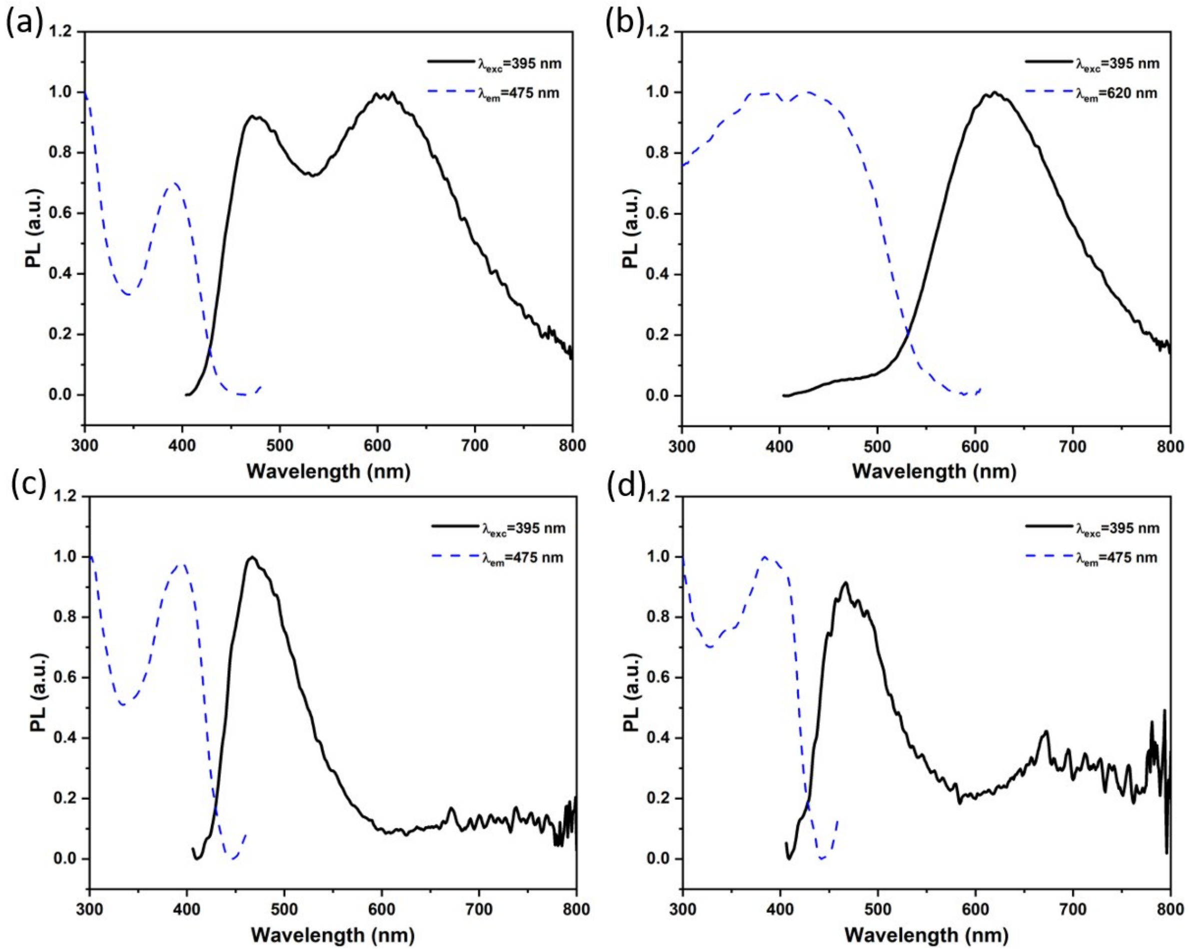

Figure S24. Fluorescence emission and excitation spectra of **Bpy-sp²c-P** in MeCN (a) and MeCN/TEOA (30/1) (b) and **Re-Bpy-sp²c-P** in MeCN (c) and MeCN/TEOA (30/1) (d).

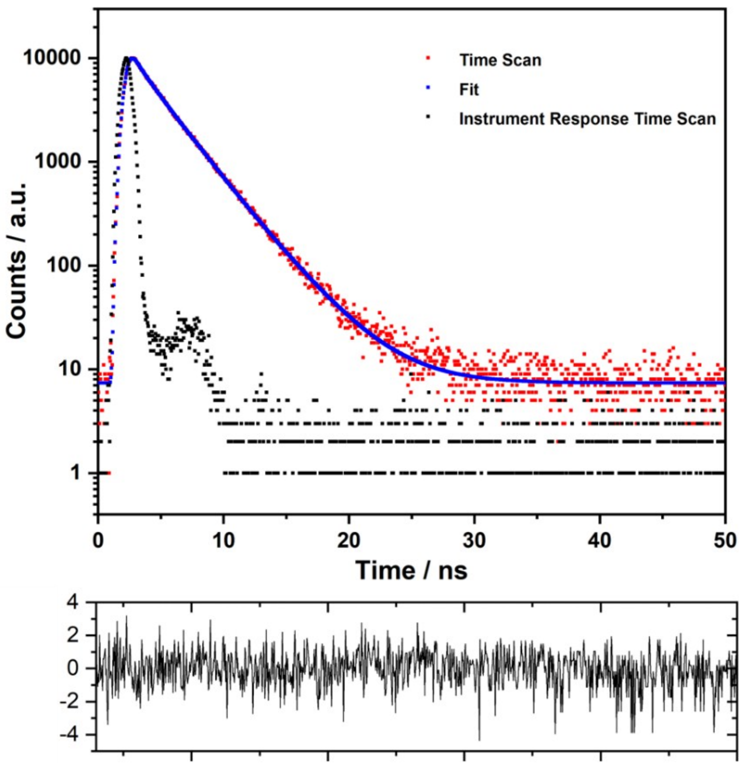

Figure S25. Fluorescence life-time decays of **Bpy-sp²c-COF** in acetonitrile purged with CO_2 (λ_{exc} = 405 nm, λ_{em} = 475 nm).

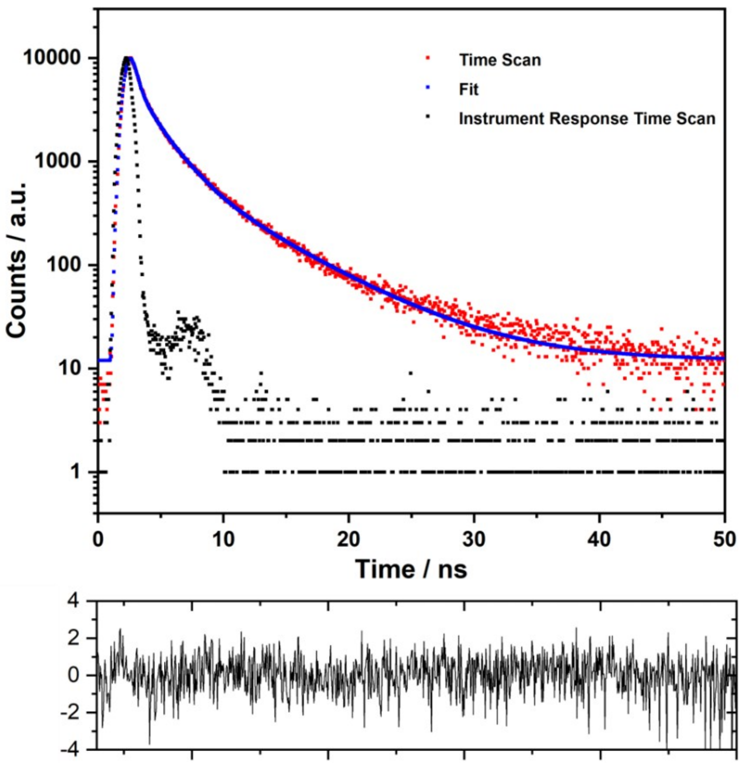

Figure S26. Fluorescence life-time decays of **Bpy-sp²c-COF** in acetonitrile purged with CO_2 (λ_{exc} = 405 nm, λ_{em} = 640 nm).

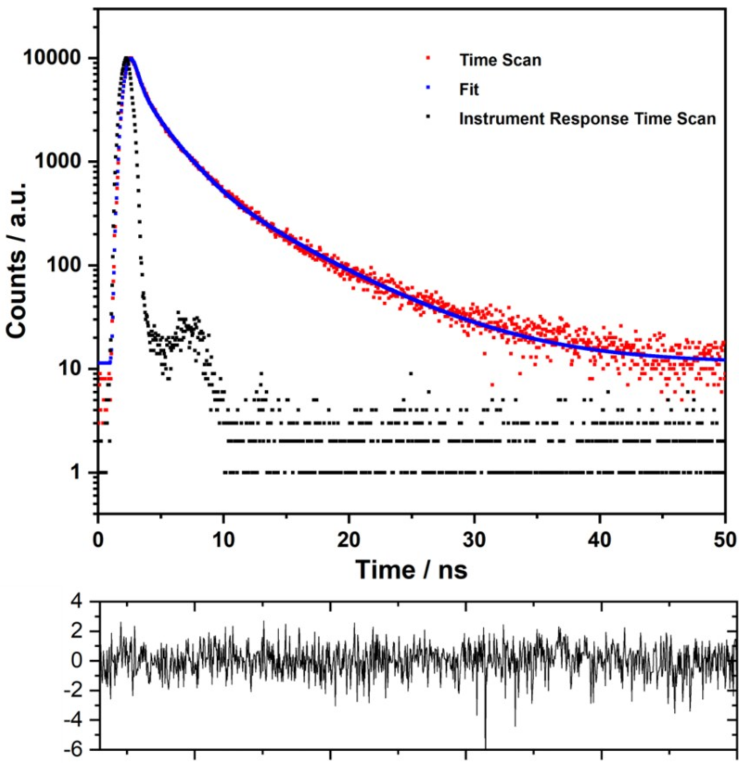

Figure S27. Fluorescence life-time decays of **Bpy-sp²c-COF** in acetonitrile and triethanolamine (30/1) mixture purged with CO_2 (λ_{exc} = 405 nm, λ_{em} = 620 nm).

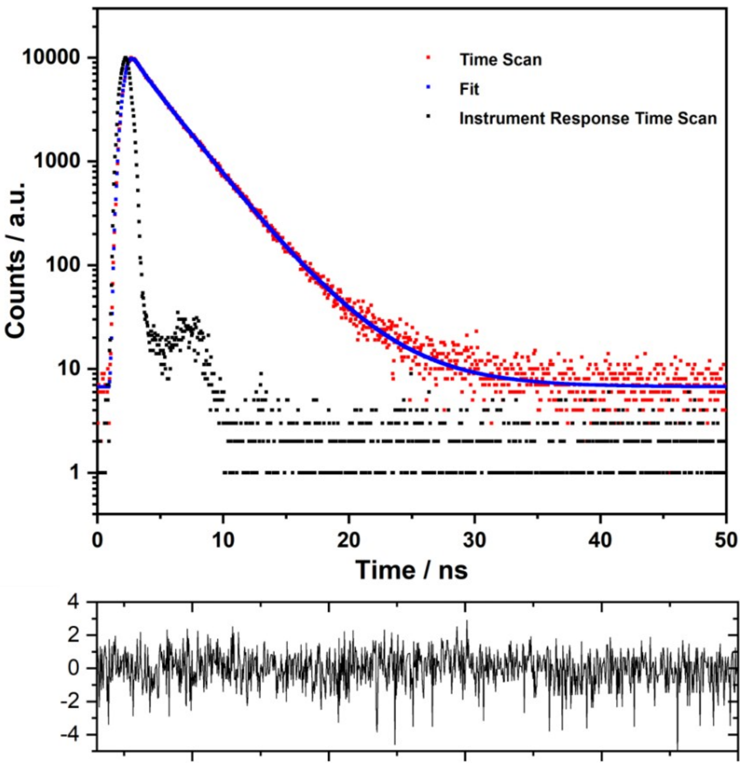

Figure S28. Fluorescence life-time decays of **Re-Bpy-sp²c-COF** in acetonitrile purged with CO_2 (λ_{exc} = 405 nm, λ_{em} = 475 nm).

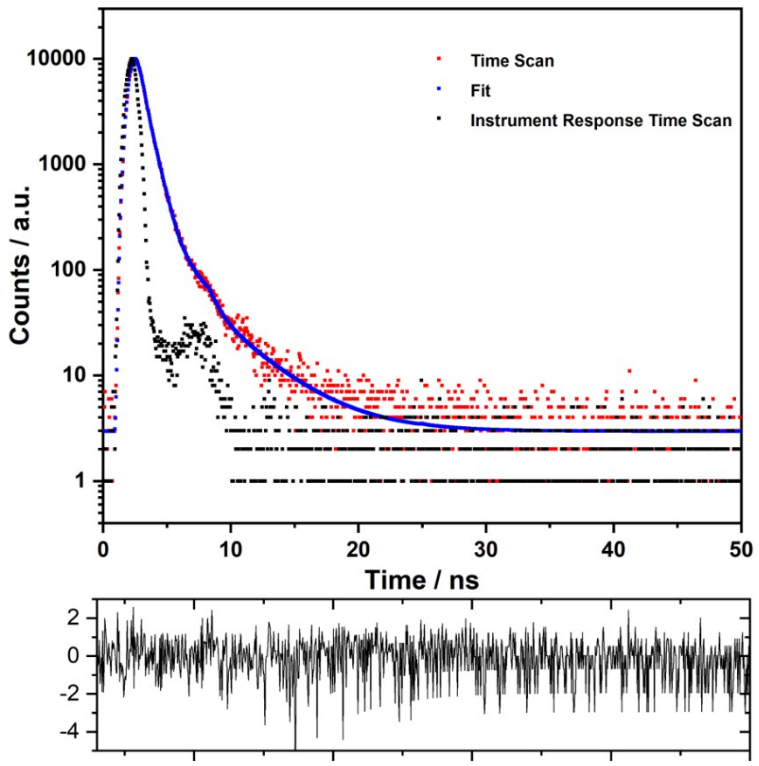

Figure S29. Fluorescence life-time decays of **Re-Bpy-sp²c-COF** in acetonitrile and triethanolamine (30/1) mixture purged with CO_2 (λ_{exc} = 405 nm, λ_{em} = 475 nm).

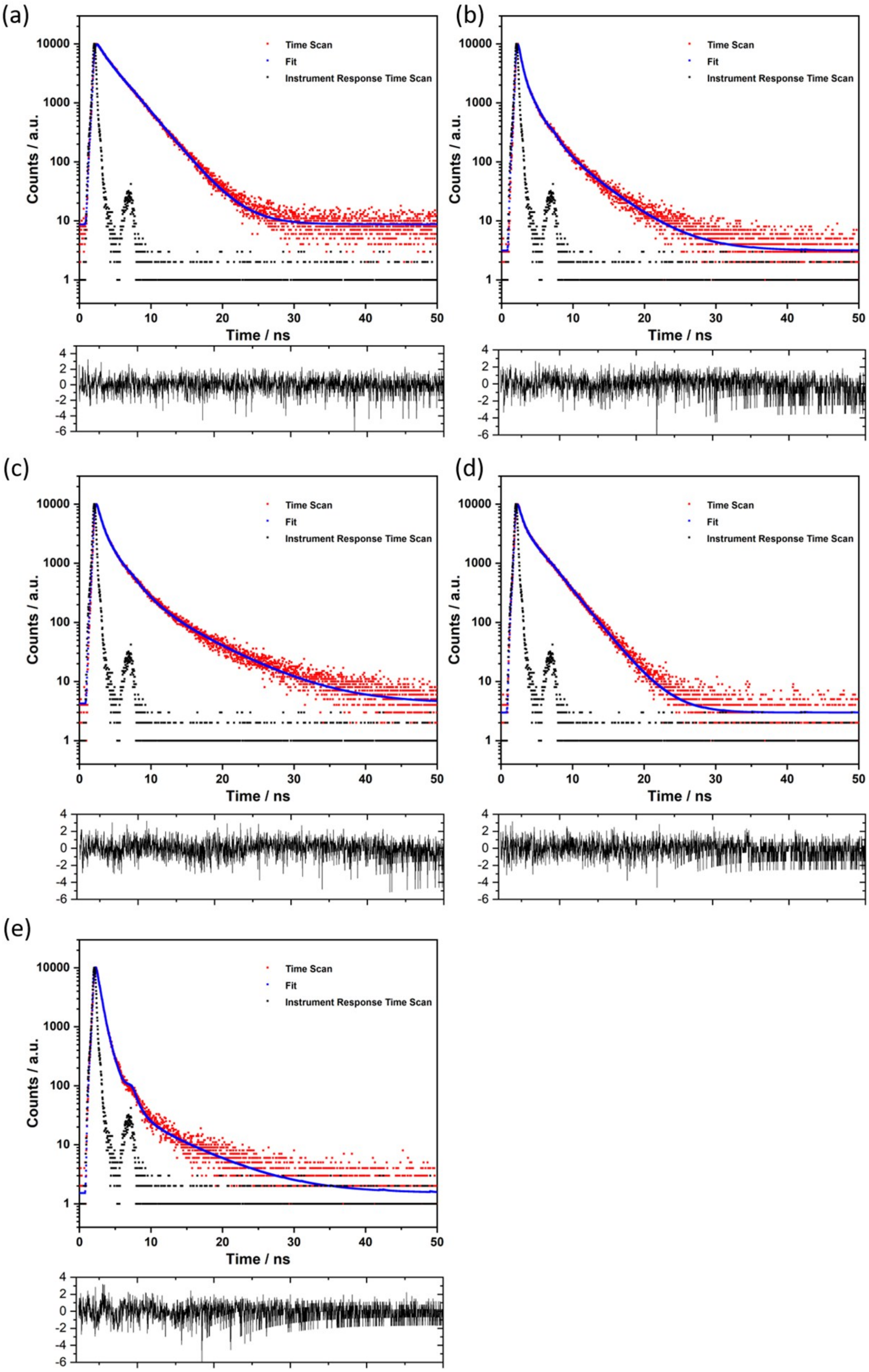

Figure S30. Fluorescence life-time decays of **Bpy-sp²c-P** in MeCN (a, b) and MeCN/TEOA (30/1) (c, d) mixture purged with CO_2 (λ_{exc} = 405 nm, λ_{em} = 475 or 620 nm) and **Re-Bpy-sp²c-P** in MeCN (d) and MeCN/TEOA (e) purged with CO_2 (λ_{exc} = 405 nm, λ_{em} = 475).

Table S1. Fluorescence life-time measurements.

**Materials**	**λ_{em} / nm**	**τ_1 / ns**	**B_1 / %**	**τ_2 / ns**	**B_2 / %**	**τ_3 / ns**	**B_3 / %**	**χ^2**	**τ_{AVG}**
**Bpy-sp^{2}c-COF[a]**	475	0.56	5.11	2.55	84.22	4.16	10.66	1.18	2.62
**Bpy-sp^{2}c-COF[b]**	475	0.06	9.16	1.95	31.27	3.13	59.57	1.25	2.48
**Bpy-sp^{2}c-COF[a]**	640	0.64	34.16	2.56	46.82	7.5	19.02	1.19	2.84
**Bpy-sp^{2}c-COF[b]**	640	0.66	38.23	2.48	42.59	7.34	19.18	1.19	2.72
**Bpy-sp^{2}c-COF[c]**	475	0.15	16.42	0.55	60.10	1.19	18.48	1.20	0.60
**Bpy-sp^{2}c-COF[d]**	475	0.08	6.14	0.66	87.52	2.20	6.34	1.02	0.72
**Bpy-sp^{2}c-COF[c]**	620	0.79	35.05	2.74	46.03	7.94	18.93	1.19	3.04
**Bpy-sp^{2}c-COF[d]**	620	0.72	33.59	2.57	46.47	7.54	19.94	1.16	2.94
**Re-Bpy-sp^{2}c-COF[a]**	475	0.14	5.90	1.98	44.30	3.09	49.80	1.39	2.42
**Re-Bpy-sp^{2}c-COF[b]**	475	0.25	4.89	2.57	78.73	4.26	16.38	1.25	2.73
**Re-Bpy-sp^{2}c-COF[c]**	475	0.22	28.82	0.75	67.21	3.96	3.97	1.29	0.72
**Re-Bpy-sp^{2}c-COF[d]**	475	0.23	26.95	0.77	68.78	3.83	4.27	1.33	0.76
**Bpy-sp^{2}c-P[a]**	475	0.33	7.65	2.24	55.04	3.23	37.31	1.31	2.47
**Bpy-sp^{2}c-P[b]**	475	0.41	4.94	2.57	65.68	3.67	29.37	1.11	2.79
**Bpy-sp^{2}c-P[a]**	620	0.36	41.04	1.42	43.42	4.39	15.55	1.37	1.44
**Bpy-sp^{2}c-P[b]**	620	0.37	40.39	1.45	42.51	4.52	17.11	1.41	1.54
**Bpy-sp^{2}c-P[c]**	620	0.52	30.22	1.94	50.57	6.24	19.22	1.31	2.34
**Bpy-sp^{2}c-P[d]**	620	0.52	30.50	1.92	50.19	6.18	19.31	1.24	2.32
**Re-Bpy-sp^{2}c-P[a]**	475	0.37	27.92	1.91	30.20	3.14	41.88	1.13	1.99
**Re-Bpy-sp^{2}c-P[b]**	475	0.39	23.77	2.43	50.10	3.60	26.12	1.19	2.25
**Re-Bpy-sp^{2}c-P[c]**	475	0.38	66.06	0.99	31.10	5.25	2.85	1.01	0.71
**Re-Bpy-sp^{2}c-P[d]**	475	0.38	57.86	0.98	38.00	6.13	4.15	1.13	0.85

[a] Acetonitrile purged with N_2.

[b] Acetonitrile purged with CO_2.

[c] Acetonitrile and triethanolamine (30/1) purged with N_2.

[d] Acetonitrile and triethanolamine (30/1) purged with CO_2.

[e] Fluorescence life-times obtained upon excitation at λ_{exc} = 405 nm with a laser and observed at λ_{em} = 475, 640 nm. Fluorescence life-times in water suspension obtained from fitting time-correlated single photon counting decays to a sum of three exponentials, which yield τ_1, τ_2, and τ_3 according to $\sum_{i=1}^{n}(A + B_i\exp(-\frac{t}{\tau_i}))$. τ_{AVG} is the weighted average lifetime calculated as $\sum_{i=1}^{n} B_i\tau_i$. Note that the poor χ^2 values are due to ultrafast decays for these

materials which were very similar to the instrument response.[7, 8]

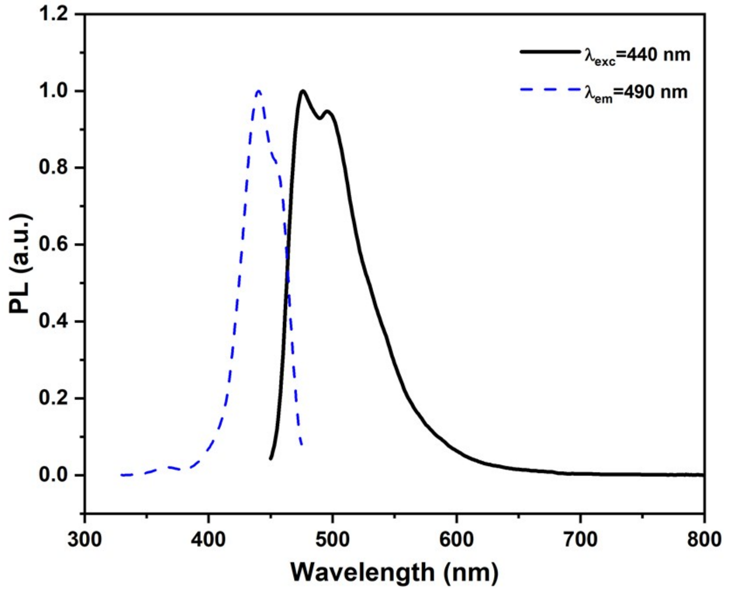

Figure S31. Fluorescence emission and excitation spectra of (Ir[dF(CF_3)ppy]$_2$(dtbpy))PF_6 in acetonitrile.

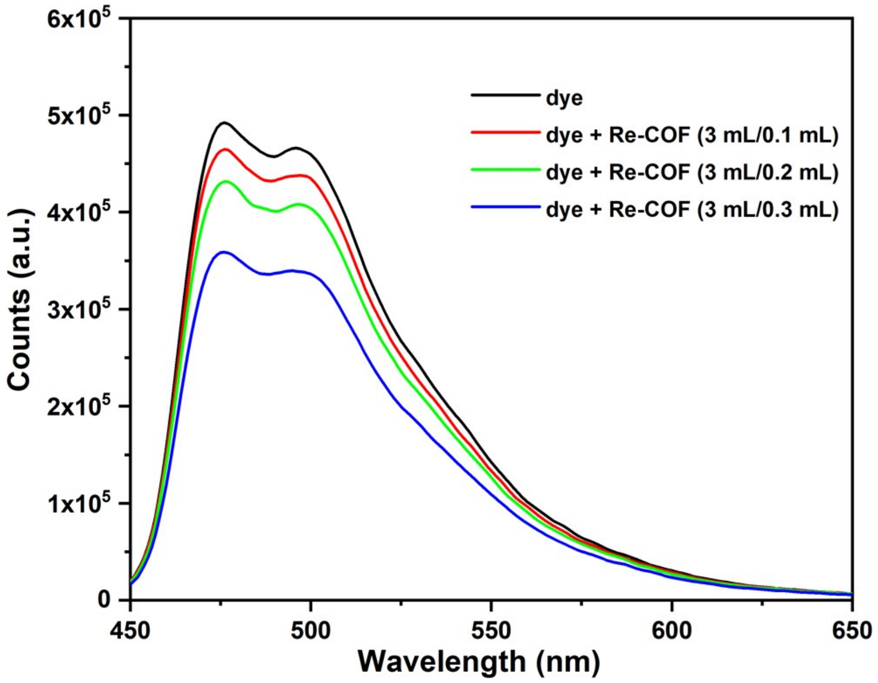

Figure S32. Fluorescence emission spectra (λ_{exc} = 440 nm) of (Ir[dF(CF_3)ppy]$_2$(dtbpy))PF_6 in acetonitrile (3 mL) and (Ir[dF(CF_3)ppy]$_2$(dtbpy))PF_6 with different amounts of **Re-Bpy-sp^{2}c-COF** and acetonitrile (1 mg in 5 mL) mixture.

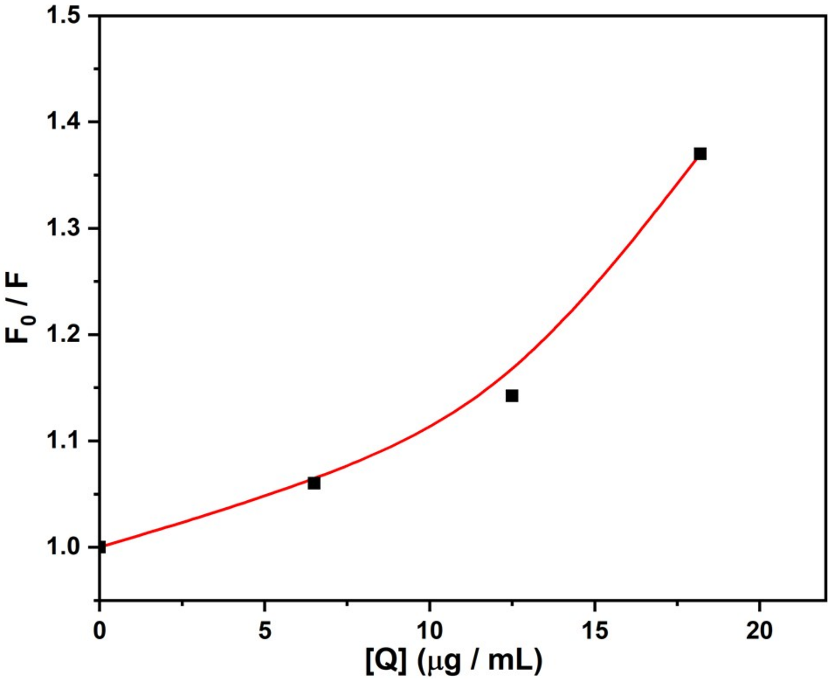

Figure S33. Stern-Volmer plots F_0/F versus Re-Bpy-sp^{2}c-COFs concentration, [Q], in acetonitrile.

Stern-Volmer plots based on fluorescence quenching intensity ratios F_0/F of dye and Re-Bpy-sp^{2}c-COF solutions are plotted in Figure S33. F_0 is the fluorescence intensity of the dye without quencher (Re-Bpy-sp^{2}c-COF) and fluorescence intensity F is for the dye with Re-Bpy-sp^{2}c-COF added in various concentrations. As shown in Figure S33, the Stern-Volmer plots show an upward (positive deviation) from a linear trend for F_0/F ratios with increasing quencher concentration, which suggesting the simultaneous presence of dynamic and static quenching.[9, 10]

12. Energy-dispersive X-ray spectroscopy

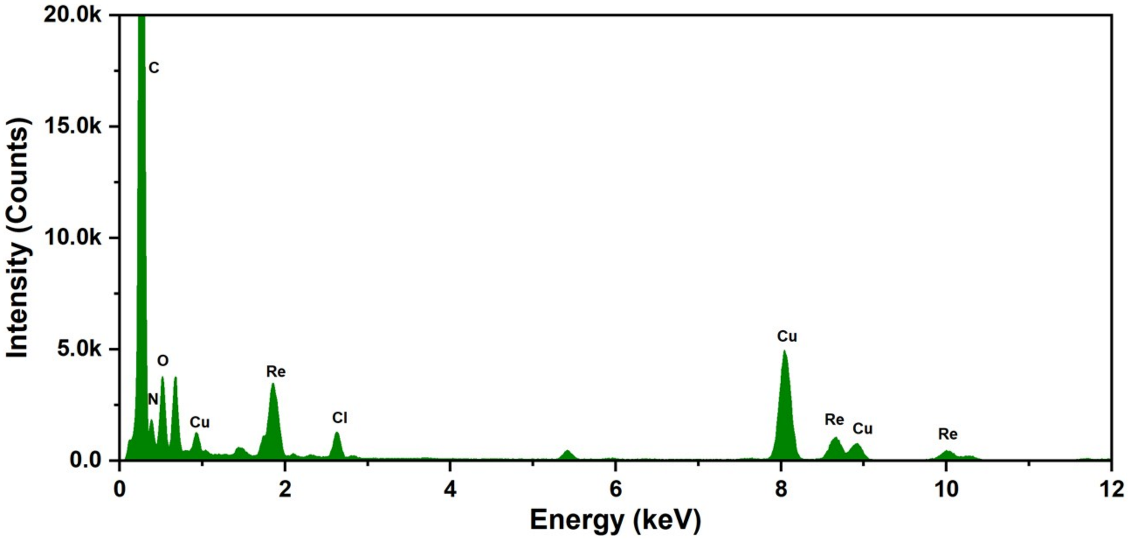

Figure S34. EDX spectrum of **Re-Bpy-sp^{2}c-COF**.

13. Wavelength-dependent CO_2 reduction experiments

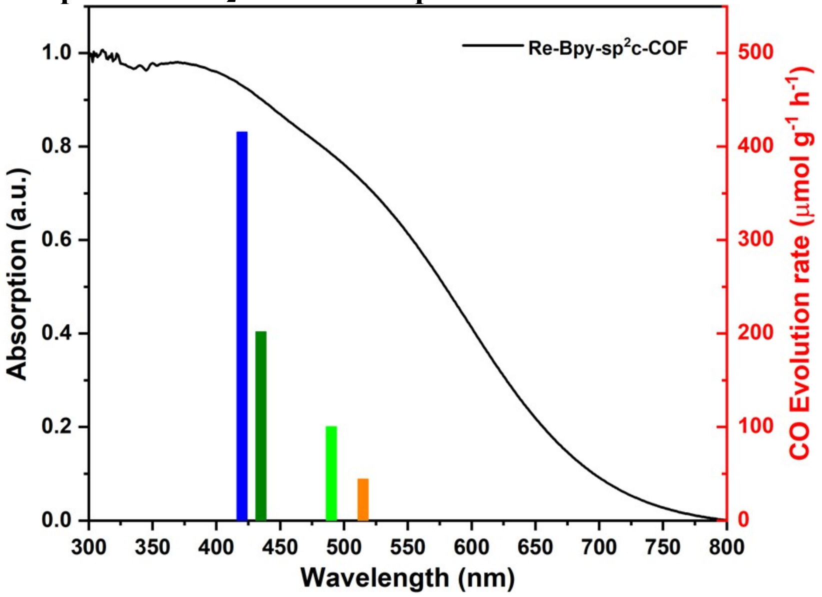

Figure S35. Wavelength dependent CO evolution experiments of **Re-Bpy-sp^{2}c-COF** (1 mg) from 5 mL MeCN/TEOA (30/1) solution under monochromatic light (± 10 nm, fwhm) in a photoreactor with path length of 5 cm at 420 nm (blue), 435 nm (deep green), 490 nm (green), and 515 nm (orange).

14. Transmission and backscattering experiments

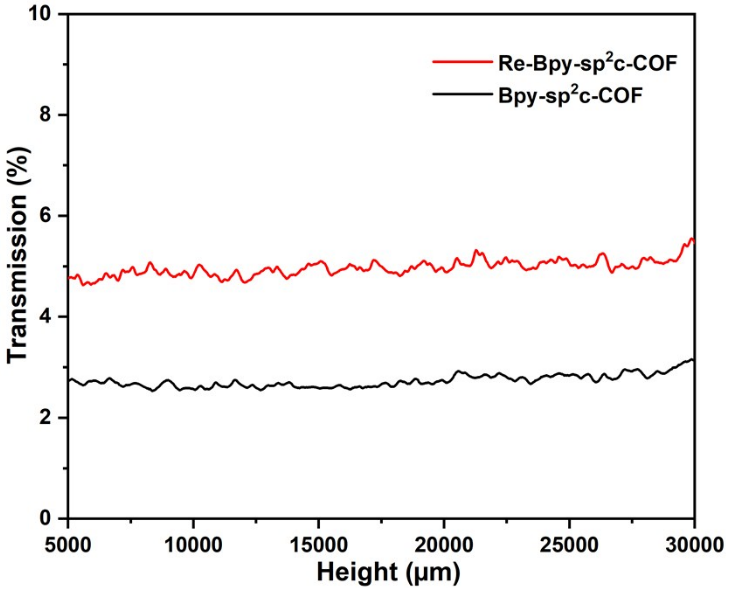

Figure S36. Transmission experiments of photocatalysts suspended in MeCN. The transmittance of the suspensions was measured with a laser scanning the height of the measurement reactor at 180° relative to the light source. The suspensions appear to be stable for the duration of the measurement as the transmission values are similar for the entire height of the measurement reactor, with in both cases low transmission values showing that the material disperses well in MeCN.

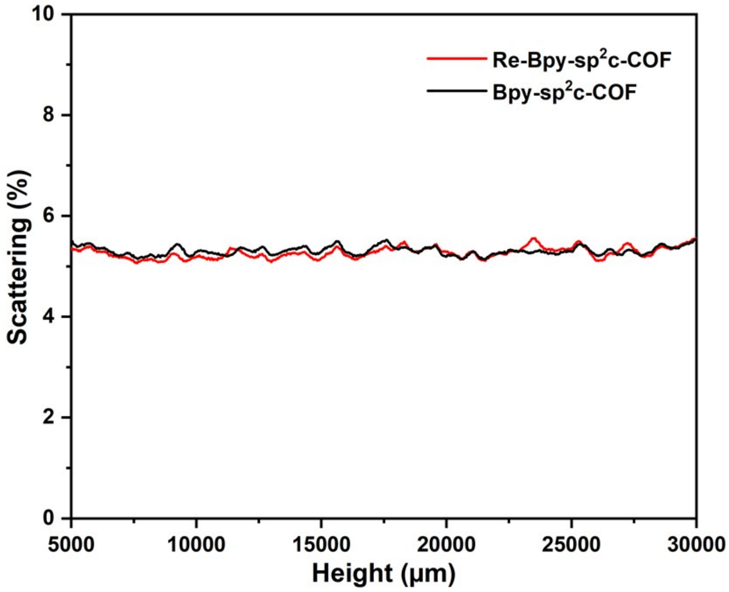

Figure S37. Backscattering experiments of photocatalysts suspended in MeCN. The backscattering of the suspensions was measured with a laser scanning the height of the measurement reactor at 45° relative to the light source. The suspensions appear to be stable for the duration of the measurement as the backscattering values are similar for the entire height of the measurement reactor.

15. Gas sorption isotherms

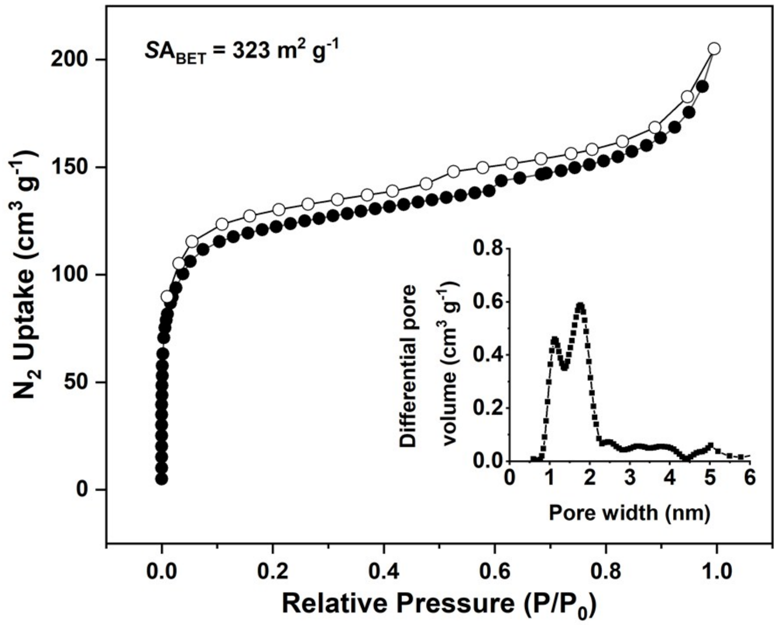

Figure S38. N_2 isotherm of **Re-Bpy-sp²c-COF** measured at 77 K (Adsorption (filled dots) and desorption (open dots)). The inset shows the calculated pore size distribution.

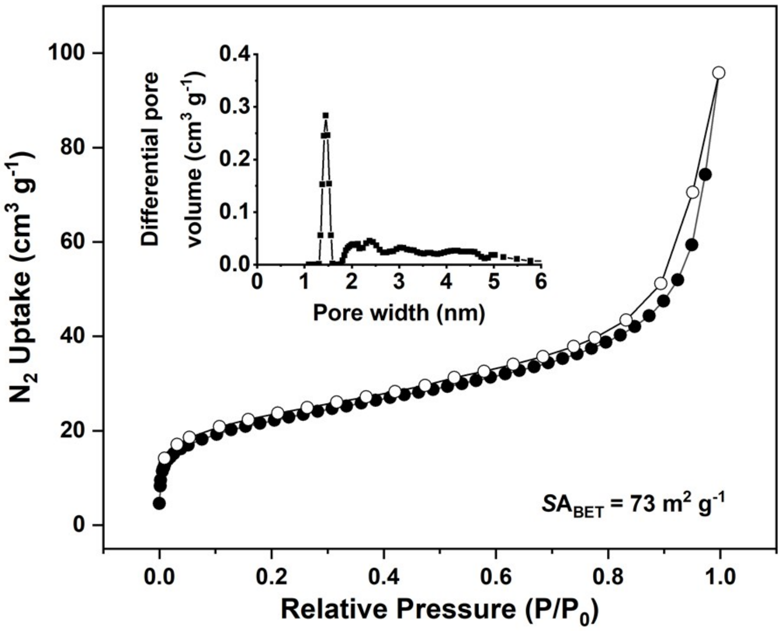

Figure S39. N_2 isotherm of **Bpy-sp²c-P** measured at 77 K (Adsorption (filled dots) and desorption (open dots)). The inset shows the calculated pore size distribution.

16. X-Ray photoelectron spectroscopy

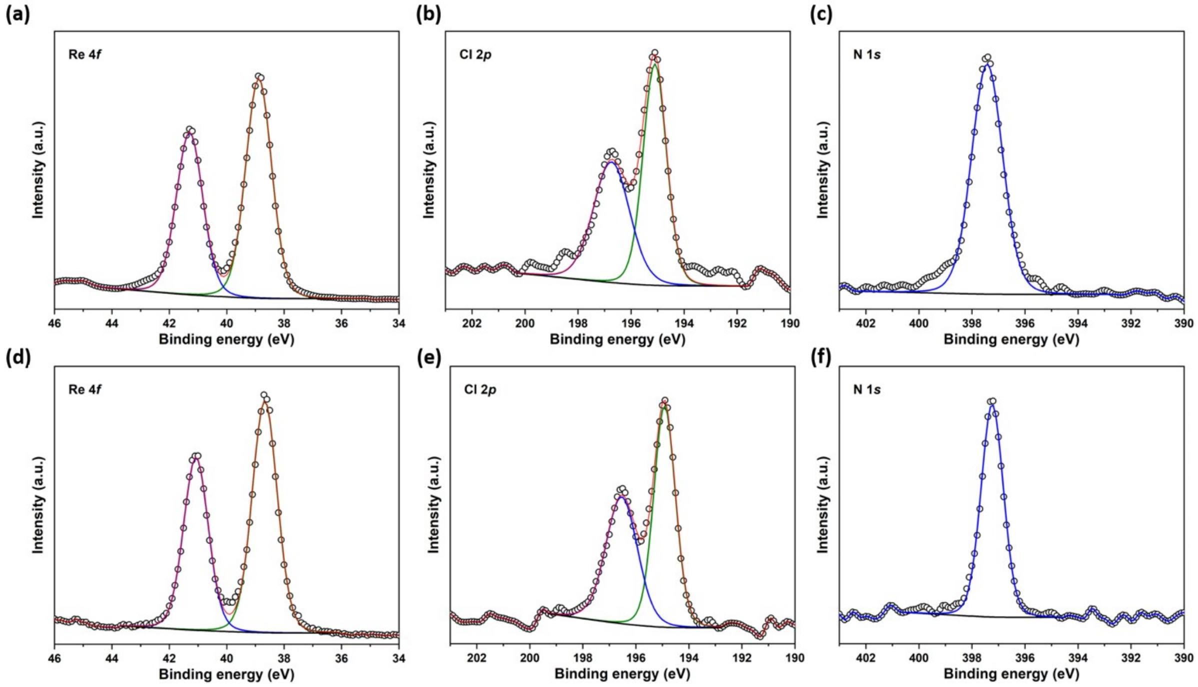

Figure S40. HR XPS analysis of Re 4f (a), Cl 2p (b) and N 2s (s) signals of **Re-Bpy-sp²c-COF** and Re 4f (d), Cl 2p (e) and N 2s (f) signals of **Re(bpy)(CO)$_3$Cl**

17. Photoelectrochemical measurements

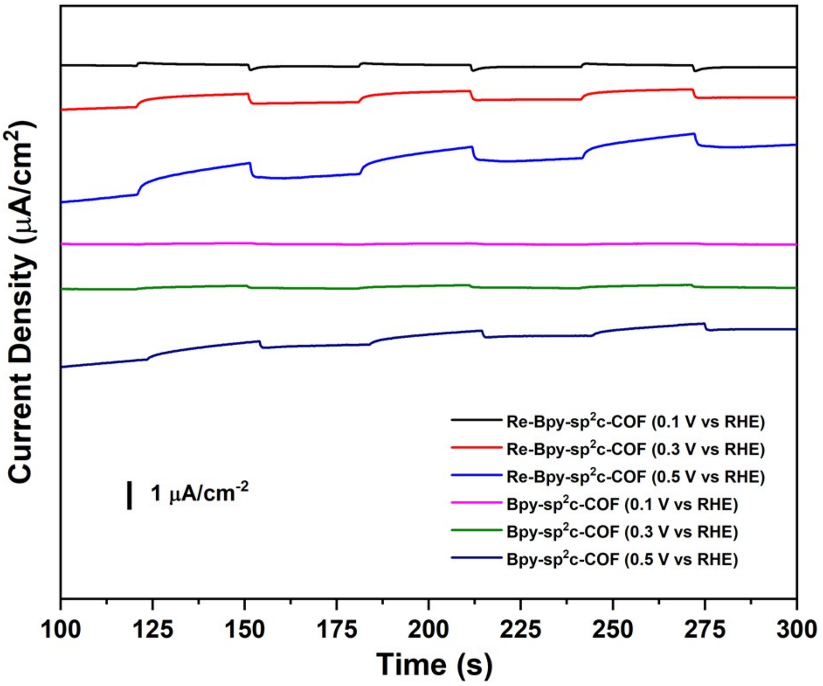

Figure S41. Transient photocurrent response at different potentials in acetonitrile vs RHE under intermittent light irradiation for **Bpy-sp^{2}c-COF** and **Re-Bpy-sp^{2}c-COF**.

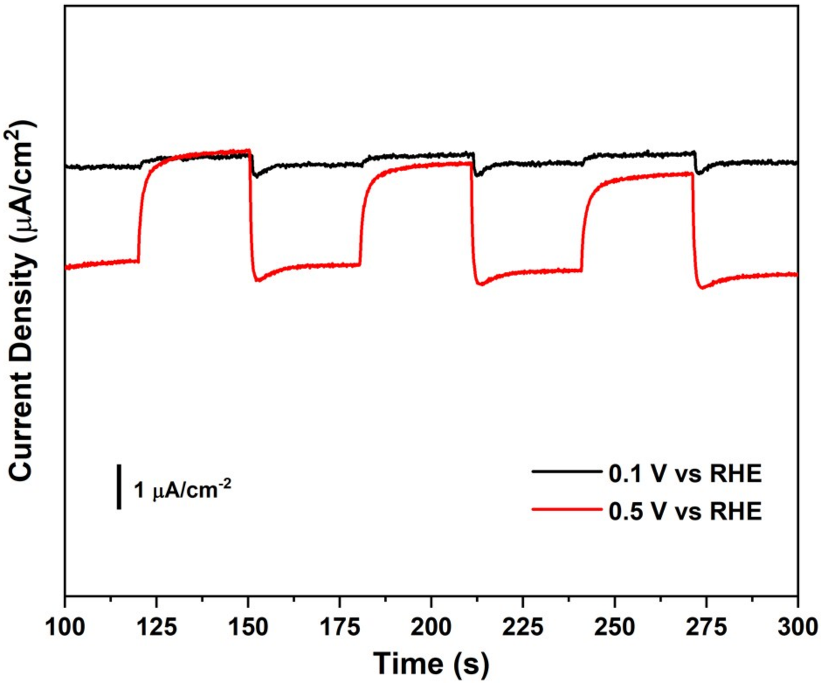

Figure S42. Transient photocurrent response at different potentials in aqueous solution vs RHE under intermittent light irradiation for **Re-Bpy-sp^{2}c-COF**.

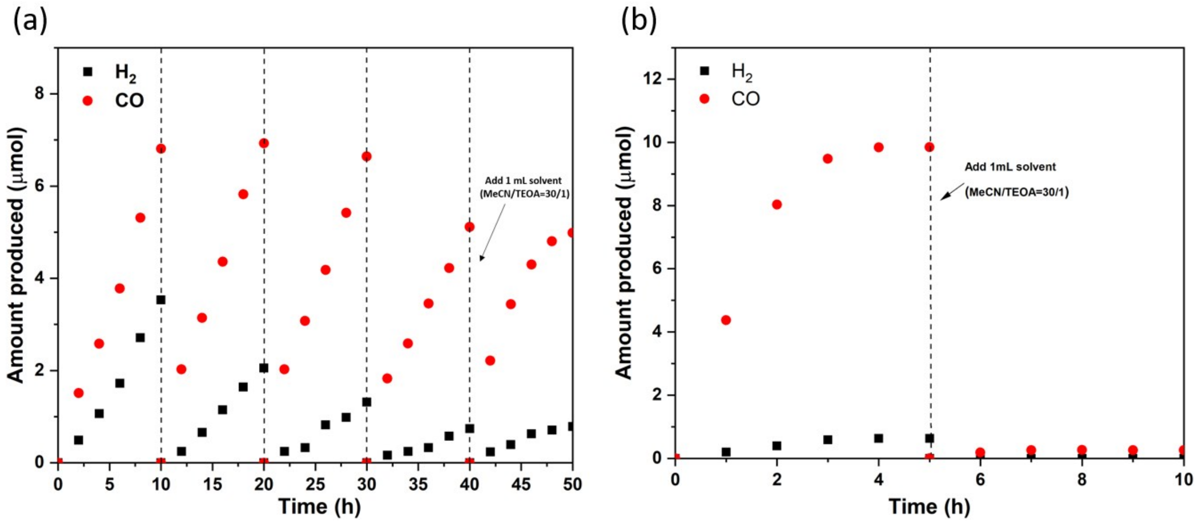

Figure S43. Stability and reusability test using **Re-Bpy-sp^{2}c-COF** (1mg) (a) and **Re(Bpy)(CO)$_3$Cl** (0.97 umol) (b) as a photocatalyst under visible light irradiation (λ > 420 nm) in 5 mL MeCN/TEOA (30/1) solvent for 50 h and 10 h.

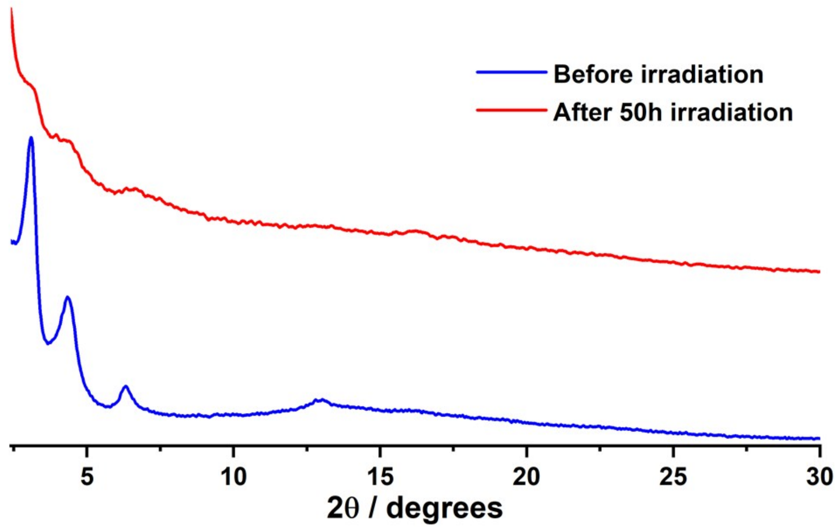

Figure S44. Experimentally observed powder X-ray diffraction pattern of **Re-Bpy-sp^{2}c-COF** before and after 50 hours irradiation

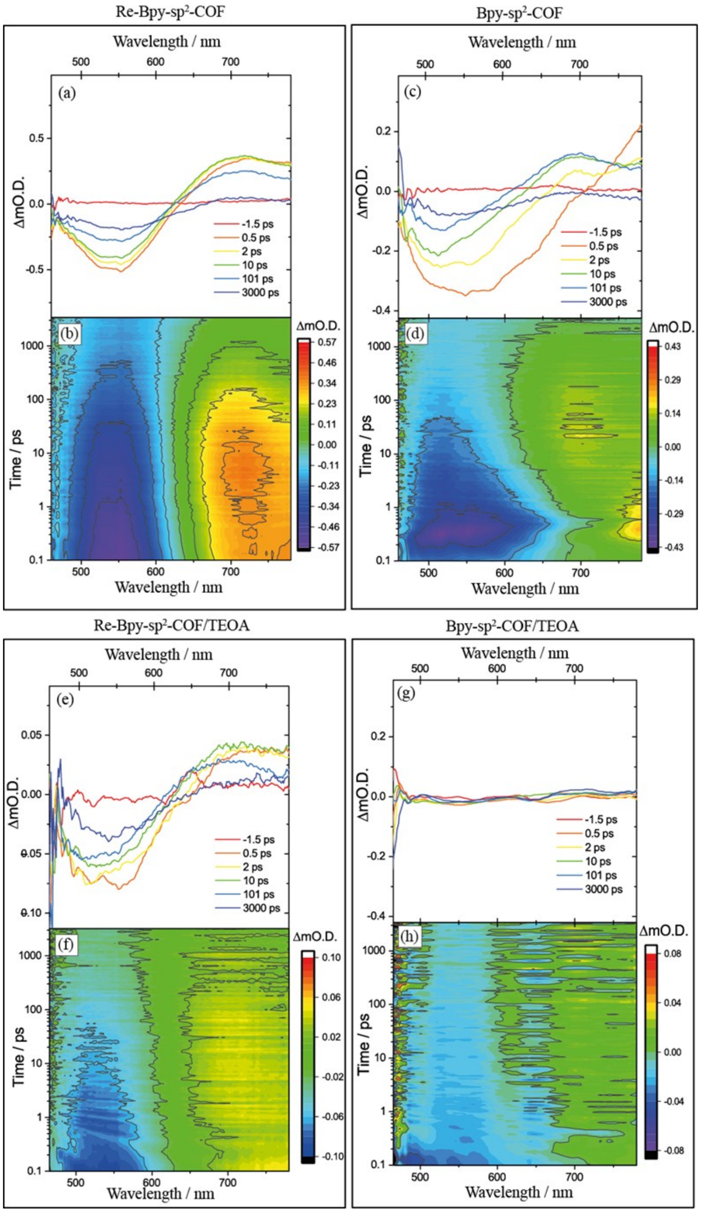

Figure S45. Transient absorption spectra of (a) Re-Bpy-sp^{2}c-COF and (c) Bpy-sp^{2}c-COF in pure acetonitrile; and (e) Re-Bpy-sp^{2}c-COF and (g) Bpy-sp^{2}c-COF in a 30:1 mixture of acetonitrile and TEOA at pump-probe time delays chosen to highlight the changing nature of the excited electronic states probed. Complete transient absorption surface probed (b) Re-Bpy-sp^{2}c-COF and (d) Bpy-sp^{2}c-COF in pure acetonitrile and (f) Re-Bpy-sp^{2}c-COF and (h) Bpy-sp^{2}c-COF in a 30:1 mixture of acetonitrile and TEOA. In the presence of TEOA we again find that the TA signals are significantly weaker with Re-Bpy-sp^{2}c-COF, figure S44. The ground state bleach of Re-Bpy-sp^{2}c-COF centred at ca. 540 nm is significantly (ca. 85%, 1 ps) diminished in the presence of TEOA even at the earliest timescales studied indicating that the TEOA is able to act as an electron-donor. Interestingly the addition of TEOA to Bpy-sp^{2}c-COF leads to only weak signals being recorded in the TA spectrum indicating that TEOA is able to reductively quench the excited states probed by with wavelengths employed in the TA experiment – in contrast to results of the TCSPC experiments.

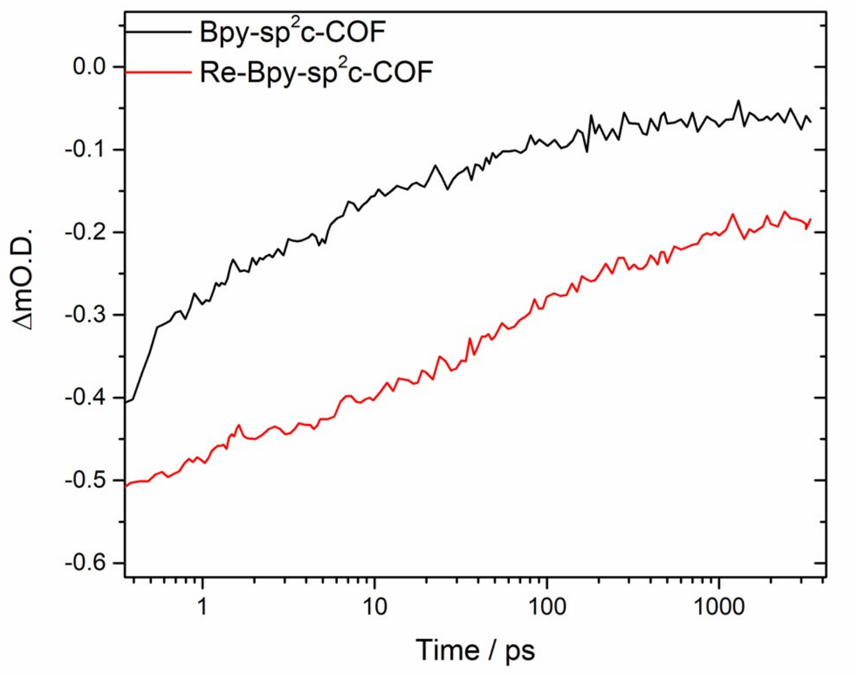

Figure S46. Transient absorption kinetics recorded at 550 nm following 400 nm excitation of the samples indicated in CH_3CN.

18. Control experiments and previously reported data

Table S2. Photocatalytic CO_2 reduction using different experimental conditions.

Entry	Photocatalyst	CO (μmol)	H_2 (μmol)	Re content (μmol)	TON (CO)	CO selectivity (%)
1[a]	Re-Bpy-sp^{2}c-COF	12.48	2.99	0.97	12.9	80.7
2[a]	Re-Bpy-sp^{2}c-COF + dye	16.80	2.64	0.97	17.3	86.4
3[a]	Bpy-sp^{2}c-COF	0.21	n.d.	0	/	/
4[b]	Re-Bpy-sp^{2}c-COF	0.18	3.42	0.97	0.2	5.0
5[c]	Re-Bpy-sp^{2}c-COF	n.d.	n.d.	0.97	/	/
6[d]	Re-Bpy-sp^{2}c-COF	n.d.	n.d.	0.97	/	/
7[e]	Re-Bpy-sp^{2}c-COF	2.32	0.76	0.97	2.4	75.2
8[b]	Re-Bpy-sp^{2}c-COF + dye	0.22	6.14	0.97	0.2	3.4
9[f]	Bpy-sp^{2}c-COF + dye	0.19	n.d.	0	/	/
10[f]	dye	n.d.	n.d.	0	/	/
11[f]	Re-Bpy-sp^{2}c-P	1.15	0.07	0.50	2.3	94.5
12[f]	Re-Bpy-sp^{2}c-P + dye	3.45	0.74	0.50	6.9	82.4
13	Bpy-sp^{2}c-P	-	0.18	0	/	-
14	Bpy-sp^{2}c-P + dye	0.19	0.18	0	/	70.4
15[g]	Re(bpy)$(CO)_3$Cl	10.03	0.67	0.97	10.3	93.8

[a] Reaction conditions: Photocatalyst (1 mg), solvent (5 mL, acetonitrile/TEOA = 30 : 1), CO_2 (1 atm.), 300 W Xe light source equipped with $\lambda > 420$ nm cut-off filter, 12 hours; [b]Ar atmosphere instead of CO_2; [c]In the dark; [d]Without TEOA; [e] Acetonitrile was replaced with dimethylformamide; [f]Solvent (5 mL, acetonitrile/TEOA = 30 : 1), CO_2 (1 atm.), 300 W Xe light source equipped with $\lambda > 420$ nm cut-off filter, 12 hours; [g]Photocatalyst (0.45 mg, 0.97 μmol), Solvent (5 mL, acetonitrile/TEOA = 30 : 1), CO_2 (1 atm.), 300 W Xe light source equipped with $\lambda > 420$ nm cutoff filter, 12 hours; Dye: (Ir[dF(CF_3)ppy]$_2$(dtbpy))PF_6; n.d.: none detected.

Table S3. Previously reported photocatalytic CO_2 reduction using COFs.

Photocatalyst	Main products and highest yield (μmol h^{-1} g^{-1})	TON	Selectivity	Reaction solvent	Irradiation condition	Reference
Re-bpy-sp^{2}c-COF	1040 (CO)	18.7 (17.5 h)	80.7% (CO)	MeCN / TEOA (30/1)	λ > 420 nm (300 W Xe light source)	This work
Re-Bpy-sp^{2}c-COF (Ir[dF(CF_3)ppy]$_2$(dtbpy))PF_6	1400 (CO)	7.2 (5 h)	86.4% (CO)	MeCN / TEOA (30/1)	λ > 420 nm (300 W Xe light source)	This work
Re-COF	~15 mmol CO/g >20h	48	98% (CO)	MeCN / TEOA (3:0.2)	λ > 420 nm (225 W Xe light source)	5
Ni-TpBpy-COF [Ru(bpy)$_3$]Cl_2	966 (CO)	13.6	96% (CO)	MeCN / H_2O / TEOA (3/1/1)	λ ≥ 420 nm (300 W Xe light source)	11
Re-TpBpy-COF	270 (CO)	-	-	MeCN / H_2O (10/1.8 mL), 0.1 M TEOA	λ > 390 nm (200 W Xe light source)	12
DQTP-COF-Co [Ru(bpy)$_3$]Cl_2	1020 (CO)	2.18	59.4% (CO)	MeCN / TEOA (4/1)	λ ≥ 420 nm (300 W Xe light source)	13
ACOF-1	0.36 (CH_3OH)	-	-	CO_2 and H_2O (0.4 MPa, 80 °C)	800 nm ≥ λ ≥ 420 nm (500 W Xe light source)	14
N_3-COF	0.57 (CH_3OH)	-	-	CO_2 and H_2O (0.4 MPa, 80 °C)	800 nm ≥ λ ≥ 420 nm (500 W Xe light source)	14

19. Density functional theory (DFT) and time-dependent DFT (TD-DFT) calculations

Representative molecular fragments (Figure S47) of the COFs studied here were calculated for their standard reduction potentials of half-reactions for free electrons/holes and excitons, using density functional theory (DFT) and time-dependent DFT (TD-DFT). The CAM-B3LYP density functional was used for all the DFT and TD-DFT calculations, together with the Def2-SVP basis set, using the Gaussian 16 software. Vertical reduction potentials (*i.e.*, IP and EA) and vertical exciton potentials (*i.e.*, IP* and EA*) were calculated using the geometry optimized in the ground state, which had been confirmed to be a true minimum by a frequency calculation.

For calculations of excited-state properties (Tables S4 and S5), S1 optimizations were first carried out using the Tamm–Dancoff approximation as this is more robust than full TD-DFT away from the ground-state geometry. Single-point, full TD-DFT calculations were then performed to obtain all necessary information for the electron excitation analyses using Multiwfn.[15] The effect of solvation by acetonitrile was accounted for by using the PCM/SMD solvation model.

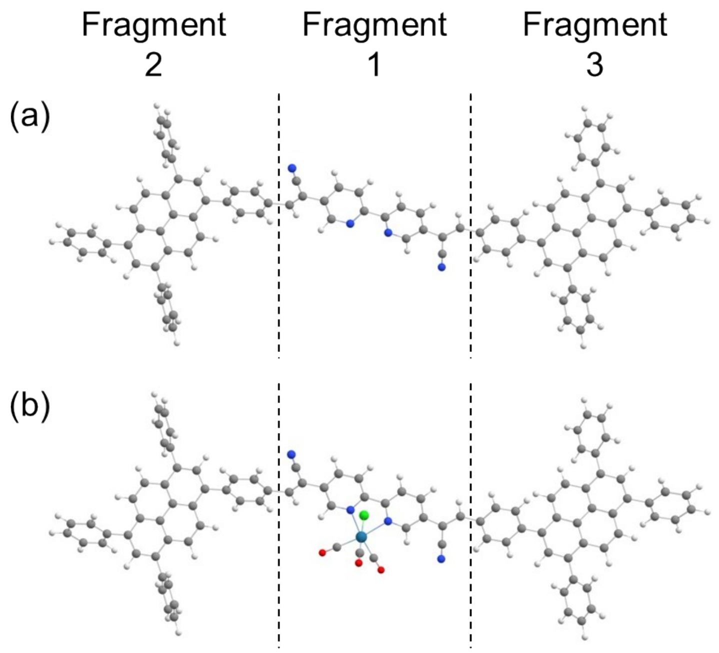

Figure S47. Representative molecular models (a) Bpy-sp^{2}c(L) and (b) Re-Bpy-sp^{2}c(L) of Bpy-sp^{2}c-COF and Re-Bpy-sp^{2}c-COF, respectively, together with fragment definition for inter-fragment charge transfer calculations.

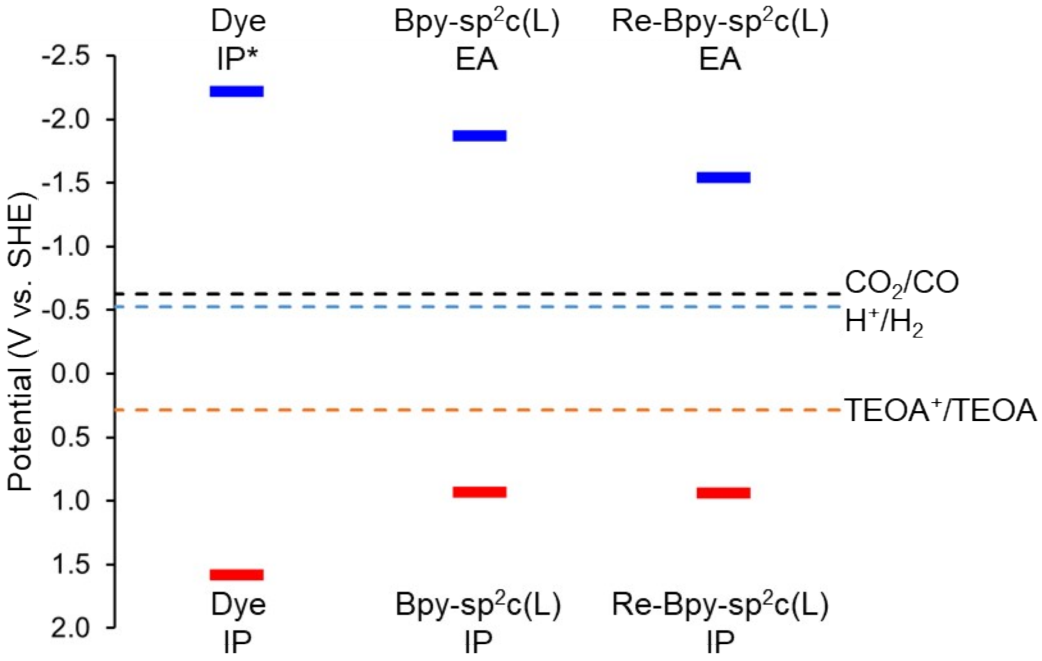

Figure S48. (TD-)CAM-B3LYP calculated IP, EA, and IP* potentials of (Ir[dF(CF_3)ppy]$_2$(dtbpy))$^-$ (dey), Bpy-sp^{2}c(L) and Re-Bpy-sp^{2}c(L). Dashed coloured lines indicate the potentials for CO_2 reduction to CO, proton reduction, and TEOA oxidation, respectively.

Table S4. Calculated TD-DFT (TD-CAM-B3LYP) excitation energies for the lowest transition (*E*), oscillator strengths (*f*), and composition in terms of molecular orbital contributions. Δ*r* is state-specific charge-transfer length.

	State	Composition [a]	*E* (eV, nm)	*f*	Δ*r* (Å) [16]
Bpy-sp^{2}c(L)	S1	84.8% H → L	2.4537 (505.29)	2.52	5.49
	S2	35.1% H-2 → L;<br>13.9% H-1 → L+1;<br>12.0% H → L+2	3.2197 (385.08)	1.50	8.72
	S3	32.8% H-1 → L+1;<br>17.7% H-1 → L+2;<br>11.0% H-2 → L	3.3683 (368.09)	1.09	10.45
Re-Bpy-sp^{2}c(L)	S1	76.0% H → L;<br>10.2% H → L+1	2.3880 (519.19)	2.64	7.61
	S2	49.8% H-2 → L	3.1047 (399.34)	0.47	6.80
	S3	30.0% H-4 → L;<br>16.7% H-2 → L	3.1889 (388.80)	0.80	6.29

a H = HOMO, L = LUMO

Table S5. Calculated inter-fragment charge transfer (in number of electrons) in the excited state (TD-CAM-B3LYP), with fragment definitions shown in Figure S47. Arrows indicate the electron transfer direction between the fragments; a negative value for the net transfer means that the electrons are transferred in the opposite direction to the one indicated by the arrow.

**Bpy-sp^{2}c(L)**					
1st Excited state					
1 → 2:	0.00	1 ← 2:	0.00	Net 1 → 2:	0.00
1 → 3:	0.12	1 ← 3:	0.25	Net 1 → 3:	-0.14
2 → 3:	0.00	2 ← 3:	0.00	Net 2 → 3:	0.00
2nd Excited state					
1 → 2:	0.08	1 ← 2:	0.11	Net 1 → 2:	-0.03
1 → 3:	0.13	1 ← 3:	0.16	Net 1 → 3:	-0.02
2 → 3:	0.10	2 ← 3:	0.08	Net 2 → 3:	0.01
3rd Excited state					
1 → 2:	0.07	1 ← 2:	0.13	Net 1 → 2:	-0.06
1 → 3:	0.03	1 ← 3:	0.05	Net 1 → 3:	-0.02
2 → 3:	0.13	2 ← 3:	0.13	Net 2 → 3:	0.00
**Re-Bpy-sp^{2}c(L)**					
1st Excited state					
1 → 2:	0.00	1 ← 2:	0.00	Net 1 → 2:	0.00
1 → 3:	0.11	1 ← 3:	0.31	Net 1 → 3:	-0.20
2 → 3:	0.00	2 ← 3:	0.00	Net 2 → 3:	0.00
2nd Excited state					
1 → 2:	0.05	1 ← 2:	0.07	Net 1 → 2:	-0.01
1 → 3:	0.15	1 ← 3:	0.13	Net 1 → 3:	0.02
2 → 3:	0.02	2 ← 3:	0.01	Net 2 → 3:	0.01
3rd Excited state					
1 → 2:	0.09	1 ← 2:	0.13	Net 1 → 2:	-0.03
1 → 3:	0.12	1 ← 3:	0.12	Net 1 → 3:	0.01
2 → 3:	0.04	2 ← 3:	0.03	Net 2 → 3:	0.01

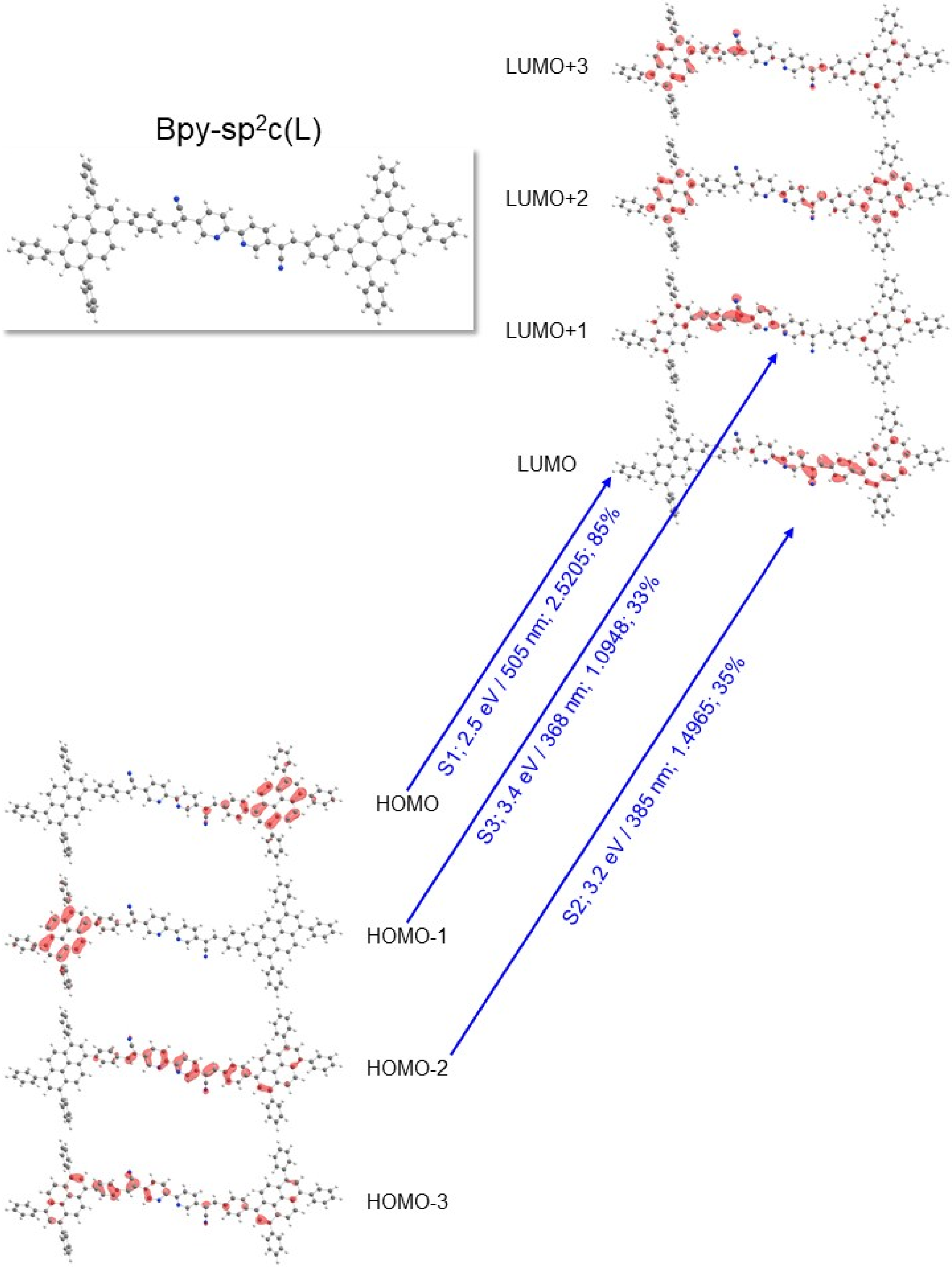

Figure S49. The frontier orbitals of Bpy-sp^{2}c(L) in the excited state (TD-CAM-B3LYP/Def2SVP); isodensity = 0.03 a.u. The major contribution to each of the first three electronic excitations is indicated by the arrow, together with the state, excitation energy (eV, nm), oscillator strength and percentage of the transition.

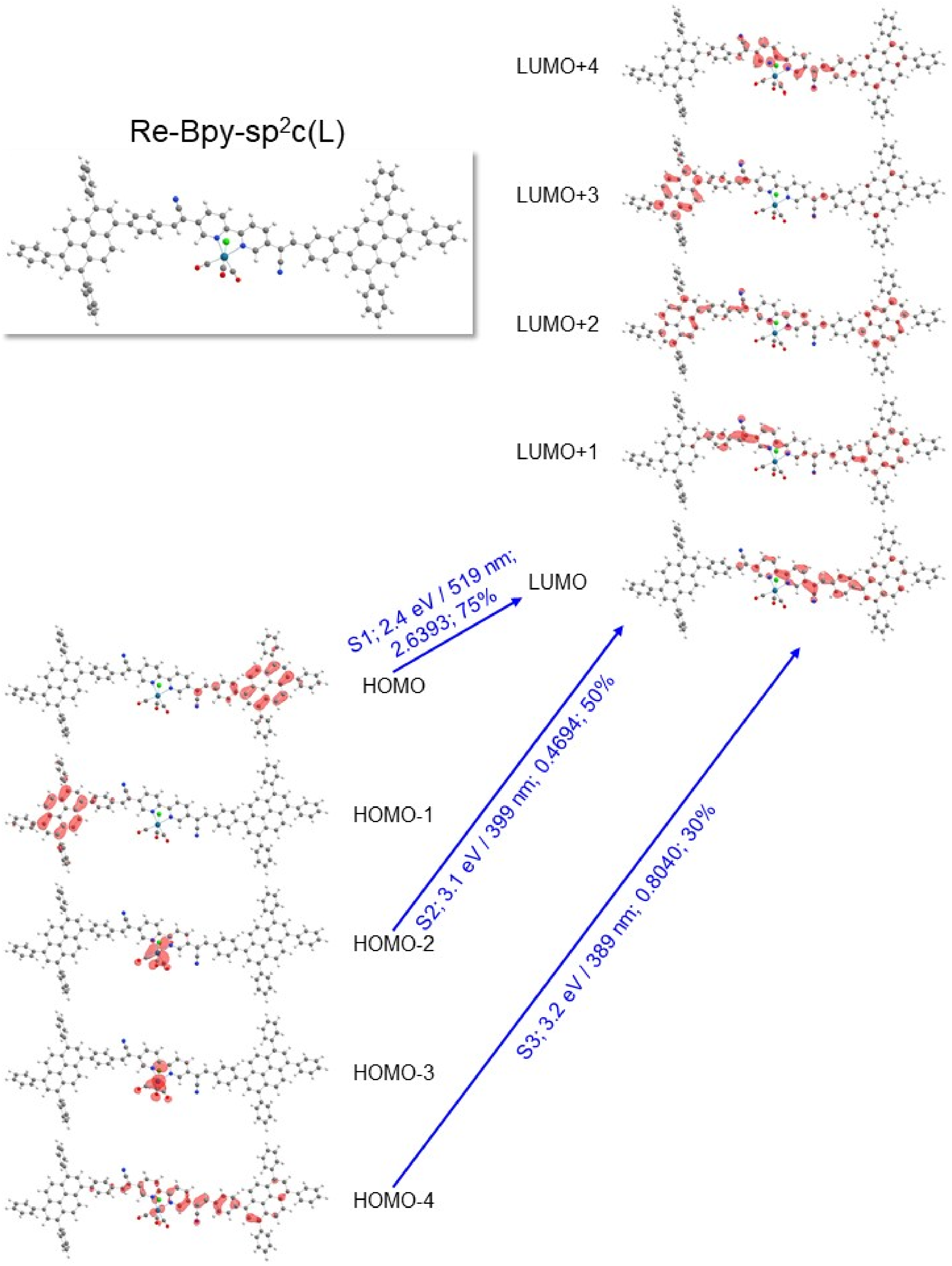

Figure S50. The frontier orbitals of Re-Bpy-sp^{2}c(L) in the excited state (TD-CAM-B3LYP/Def2SVP); isodensity = 0.03 a.u. The major contribution to each of the first three electronic excitations is indicated by the arrow, together with the state, excitation energy (eV, nm), oscillator strength and percentage of the transition.

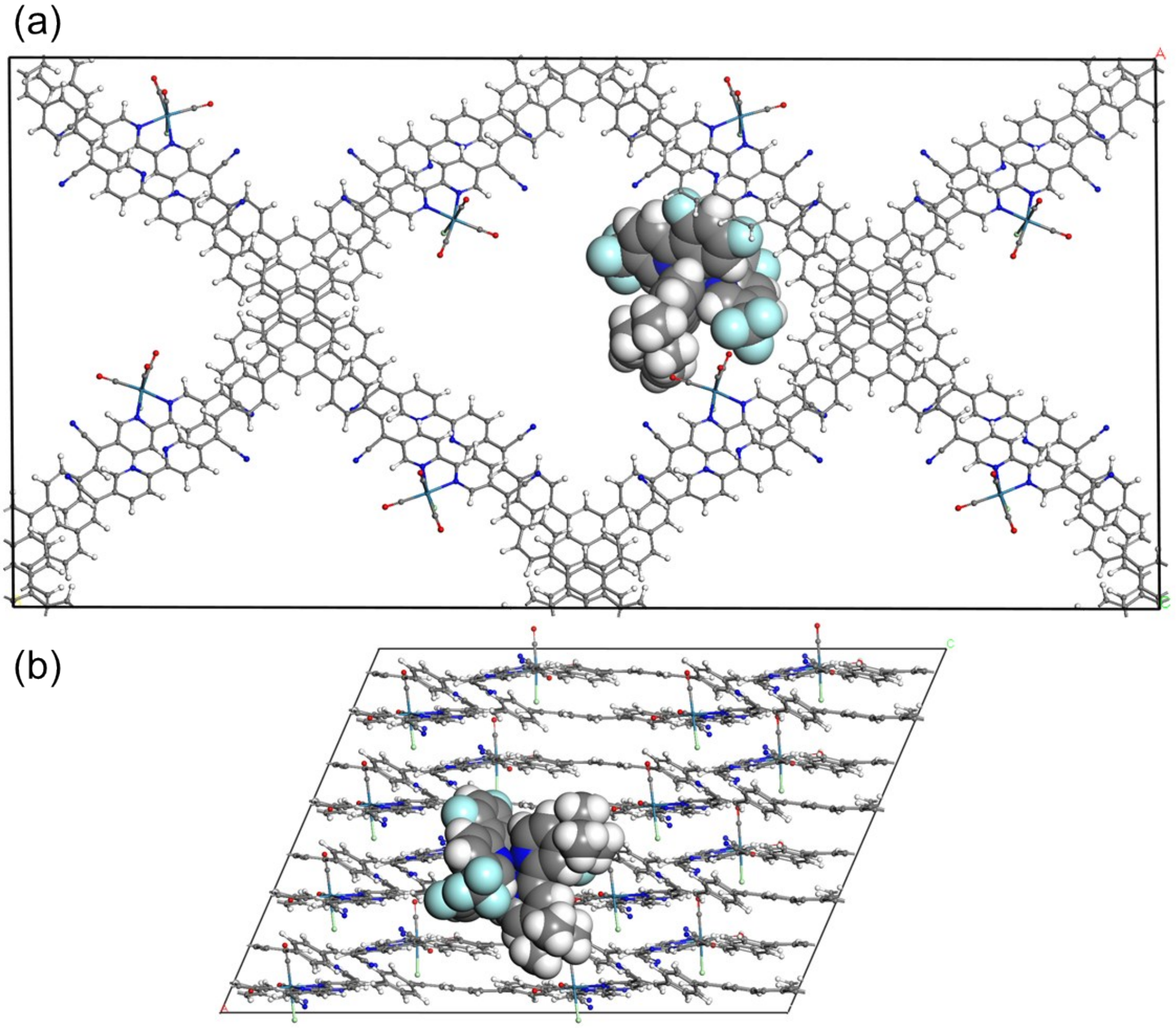

Figure S51. Figure showing simulated positioning of the $(Ir[dF(CF_3)ppy]_2(dtbpy))^+$ dye inside the Re-Bpy-sp^{2}c-COF crystal structure, viewed parallel to the pore channel along the crystallographic c-axis (a) and perpendicular to the layers (b). This model shows just one possible low-energy adsorption site in the COF pore channel, mostly to highlight the relative size of the dye molecule with respect to the channels of the COF (the channel is large enough to accommodate multiple dye molecules).

References

1. Liao, L. Y.; Kong, X. R.; Duan, X. F. Reductive Couplings of 2-Halopyridines without External Ligand: Phosphine-Free Nickel-Catalyzed Synthesis of Symmetrical and Unsymmetrical 2,2'-Bipyridines. *J. Org. Chem.* **2014**, *79* (2), 777-782.
2. Rabbani, M. G.; Sekizkardes, A. K.; El-Kadri, O. M.; Kaafarani, B. R.; El-Kaderi, H. M. Pyrene-Directed Growth of Nanoporous Benzimidazole-Linked Nanofibers and Their Application to Selective CO_2 Capture and Separation. *J. Mater. Chem.* **2012**, *22* (48), 25409-25417.
3. Coelho, A. A. TOPAS and TOPAS-Academic: an optimization program integrating computer algebra and crystallographic objects written in C++. *J. Appl. Crystallogr.* **2018**, *51* (1), 210–218.
4. Zhang, T.; Hou, Y.; Dzhagan, V.; Liao, Z.; Chai, G.; Löffler, M.; Olianas, D.; Milani, A.; Xu, S.; Tommasini, M.; Zahn, D.; Zheng, Z.; Zschech, E.; Jordan, R.; Feng, X. Copper-surface-mediated synthesis of acetylenic carbon-rich nanofibers for active metal-free photocathodes. *Nature communications*, **2018**, 9(1), 1140.
5. Yang, S.; Hu, W.; Zhang, X.; He, P.; Pattengale, B.; Liu, C.; Cendejas, M.; Hermans, I.; Zhang, X.; Zhang, J.; Huang, J. 2D Covalent Organic Frameworks as Intrinsic Photocatalysts for Visible Light-Driven CO_2 Reduction. *J. Am. Chem. Soc.* **2018**, *140* (44), 14614-14618.
6. Schneider, T. W.; Ertem, M. Z.; Muckerman, J. T.; Angeles-Boza, A. M. Mechanism of Photocatalytic Reduction of CO_2 by $Re(Bpy)(CO)_3Cl$ from Differences in Carbon Isotope Discrimination. *ACS Catal.* **2016**, *6* (8), 5473-5481.
7. Wang, X.; Chen, L.; Chong, S. Y.; Little, M. A.; Wu, Y.; Zhu, W. H.; Clowes, R.; Yan, Y.; Zwijnenburg, M. A.; Sprick, R. S.; Cooper, A. I. Sulfone-Containing Covalent Organic Frameworks for Photocatalytic Hydrogen Evolution from Water. *Nat. Chem.* **2018**, *10* (12), 1180–1189.
8. Sprick, R. S.; Bai, Y.; Guilbert, A. A. Y.; Zbiri, M.; Aitchison, C. M.; Wilbraham, L.; Yan, Y.; Woods, D. J.; Zwijnenburg, M. A.; Cooper, A. I. Photocatalytic Hydrogen Evolution from Water Using Fluorene and Dibenzothiophene Sulfone-Conjugated Microporous and Linear Polymers. *Chem. Mater.* **2019**, *31* (2), 305-313.
9. Gao, C.; Chen, S.; Wang, Y.; Wang, J.; Zheng, X.; Zhu, J.; Song, L.; Zhang, W., Xiong, Y. Heterogeneous single-atom catalyst for visible-light-driven high-turnover CO_2 reduction: the role of electron transfer. *Advanced Materials*, **2018**, *30* (13), 1704624.
10. Chen, J. K.; Yang, S. M.; Li, B. H.; Lin, C. H.; Lee, S. Fluorescence quenching investigation of methyl red adsorption on aluminum-based metal–organic frameworks. *Langmuir*, **2018**, *34*(4), 1441-1446.
11. Zhong, W.; Sa, R.; Li, L.; He, Y.; Li, L.; Bi, J.; Zhuang, Z.; Yu, Y.; Zou, Z. A Covalent Organic Framework Bearing Single Ni Sites as a Synergistic Photocatalyst for Selective Photoreduction of CO_2 to CO. *J. Am. Chem. Soc.* **2019**, *141*, 7615–7621.
12. Li, S. Y.; Meng, S.; Zou, X.; El-Roz, M.; Telegeev, I.; Thili, O.; Liu, T. X.; Zhu, G. Rhenium-

Functionalized Covalent Organic Framework Photocatalyst for Efficient CO_2 Reduction under Visible Light. *Microporous Mesoporous Mater.* **2019**, *285*, 195–201.

13. Lu, M.; Li, Q.; Liu, J.; Zhang, F. M.; Zhang, L.; Wang, J. L.; Kang, Z. H.; Lan, Y. Q. Installing Earth-Abundant Metal Active Centers to Covalent Organic Frameworks for Efficient Heterogeneous Photocatalytic CO_2 Reduction. *Appl. Catal. B Environ.* **2019**, *254*, 624–633.
14. Fu, Y.; Zhu, X.; Huang, L.; Zhang, X.; Zhang, F.; Zhu, W. Azine-Based Covalent Organic Frameworks as Metal-Free Visible Light Photocatalysts for CO_2 Reduction with H_2O. *Appl. Catal. B Environ.* **2018**, *239*, 46–51.
15. Lu, T.; Chen, F. Multiwfn: a multifunctional wavefunction analyzer. *Journal of computational chemistry*, **2012**, *33*(5), 580-592.
16. Guido, C. A.; Cortona, P.; Mennucci, B.; Adamo, C. On the metric of charge transfer molecular excitations: a simple chemical descriptor. *Journal of chemical theory and computation*, **2013**, *9*(7), 3118-3126.
